# miR-24 limits aortic vascular inflammation and murine abdominal aneurysm development

**DOI:** 10.1038/ncomms6214

**Published:** 2014-10-31

**Authors:** Lars Maegdefessel, Joshua M. Spin, Uwe Raaz, Suzanne M. Eken, Ryuji Toh, Junya Azuma, Matti Adam, Futoshi Nagakami, Helen M. Heymann, Ekaterina Chernugobova, Hong Jin, Joy Roy, Rebecka Hultgren, Kenneth Caidahl, Sonja Schrepfer, Anders Hamsten, Per Eriksson, Michael V. McConnell, Ronald L. Dalman, Philip S. Tsao

**Affiliations:** 1 Division of Cardiovascular Medicine, Stanford University, Falk CVRB, 300 Pasteur Drive, Stanford, California 94305, USA; 2 Center for Molecular Medicine L8:03, Department of Medicine, Karolinska Institute and University Hospital, Stockholm 17176, Sweden; 3 VA Palo Alto Health Care System, 3801 Miranda Avenue, Palo Alto, California 94304, USA; 4 Center of Medical Innovation and Translational Research, Department of Clinical Gene Therapy, Osaka University, 2-2 Yamada-oka, Osaka 565-0871, Japan; 5 Center for Molecular Medicine L8:03, Department of Molecular Medicine and Surgery, Karolinska Institute and University Hospital, Stockholm 17176, Sweden; 6 Transplant and Stem Cell Immunology Laboratory, University Heart Center Hamburg, Martinistraße 52, Hamburg 20246, Germany; 7 Division of Vascular Surgery, Stanford University, 300 Pasteur Drive, Stanford, California 94305, USA

## Abstract

Identification and treatment of abdominal aortic aneurysm (AAA) remain among the most prominent challenges in vascular medicine. MicroRNAs (miRNAs) are crucial regulators of cardiovascular pathology and represent intriguing targets to limit AAA expansion. Here we show, by using two established murine models of AAA disease along with human aortic tissue and plasma analysis, that miR-24 is a key regulator of vascular inflammation and AAA pathology. *In vivo* and *in vitro* studies reveal chitinase 3-like 1 (Chi3l1) to be a major target and effector under the control of miR-24, regulating cytokine synthesis in macrophages as well as their survival, promoting aortic smooth muscle cell migration and cytokine production, and stimulating adhesion molecule expression in vascular endothelial cells. We further show that modulation of miR-24 alters AAA progression in animal models, and that miR-24 and CHI3L1 represent novel plasma biomarkers of AAA disease progression in humans.

## Abstract (web summary)

Abdominal aortic aneurysm (AAA) is a potentially fatal and often asymptomatic disease whose causes remain unclear. Here the authors show that a microRNA, miR-24, and its target, the glycoprotein chitinase 3-like 1, represent key regulators of AAA development.

## Graphical abstract

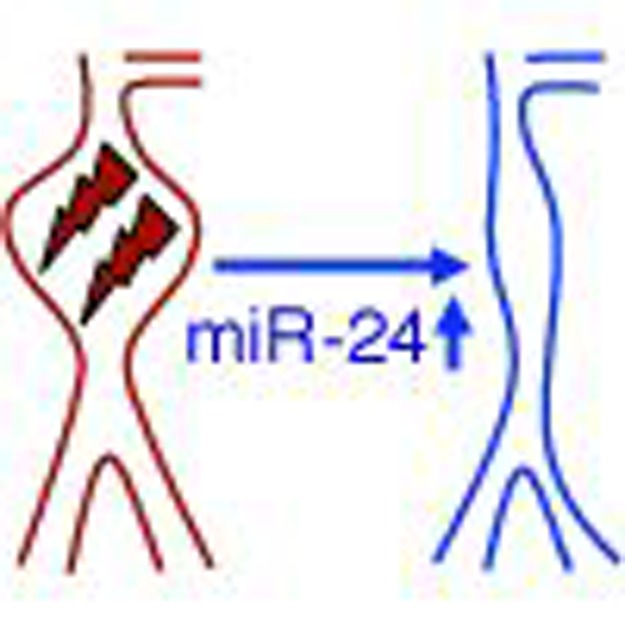

Abdominal aortic aneurysm (AAA) is a common, often asymptomatic, potentially lethal disease. No pharmacological approach has successfully decreased expansion or prevented rupture of AAA in humans^1^. microRNAs (miRNAs or miRs) are key post-transcriptional gene regulators in health and disease, typically altering the translational output of target messenger RNAs (mRNAs) by promoting degradation or preventing translation^2^. miRNA mimics and antagonists are capable of modulating entire functional networks, suggesting significant therapeutic potential^3^.

Tissue inflammation and remodelling are central elements in vascular pathogenesis and AAA expansion. Many inflammatory cell subtypes are found in human AAA tissue, macrophages being the most common^4^. In animal AAA models, macrophage accumulation in the aortic wall is one of the most consistent features from initiation to advanced aneurysm formation^1^. Further, numerous macrophage-secreted cytokines and chemokines play important roles in human AAA^5^,^6^,^7^. For the current study, we utilized miRNA and gene expression microarrays to identify novel contributors to AAA development.

We find that aortic aneurysm progression is associated with downregulation of the miR-23b-24-27b cluster in murine AAA models, with miR-24 displaying the most significant inverse regulation of its predicted targets in array profiling studies. Human AAA also display miR-24 downregulation, correlating inversely with aneurysm size. Among the most consistent and highly regulated miR-24 targets in murine AAA is a mediator/marker of inflammation: ‘chitinase 3-like 1’ (Chi3l1). We explore miR-24 regulatory mechanisms, and show that miR-24 regulates inflammation and other critical aneurysm-related processes in a CHI3L1-dependent fashion in M1-subtype macrophages, aortic smooth muscle cells (SMCs) and vascular endothelial cells. Further, we demonstrate that *in vivo* miR-24 modulation affects murine AAA progression, suggesting that miR-24 downregulation contributes to aneurysm growth. In contrast, miR-24 overexpression mitigates AAA, suggesting therapeutic potential. Additional studies suggest that miR-24 and CHI3L1 are novel plasma biomarkers of human AAA disease progression.

## Results

### miR-23b-24-27b cluster in murine AAA

**Figure 1:**
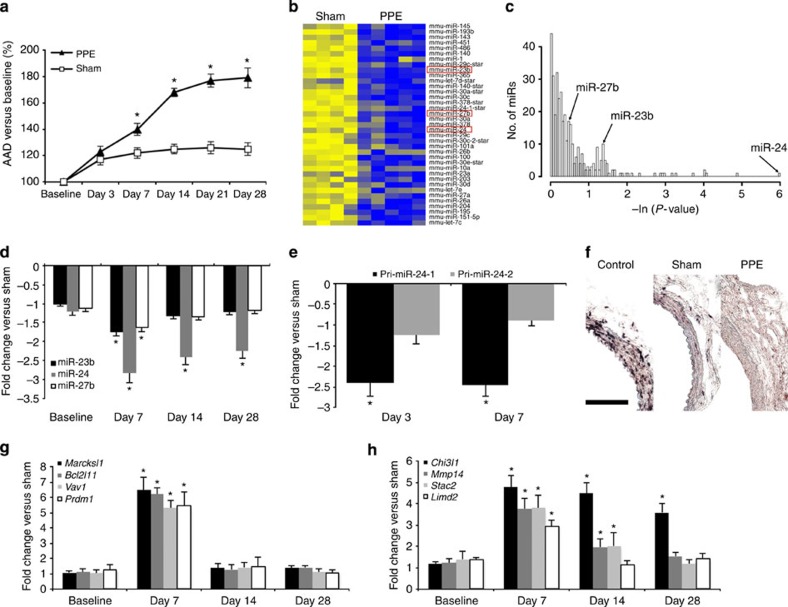
miRNAs in mouse AAA. (**a**) Abdominal aortic diameter (AAD; in % versus baseline) expansion in porcine-pancreatic-elastase (PPE)-induced AAA compared with sham-operated mice. (**b**) Significantly downregulated aortic miRNAs (miR-23b-24-27b family highlighted), 7 days after PPE induction, compared with sham. (**c**) DIANA-mirExTra histogram corresponding to (**b**), ranking downregulated miRNAs by −ln (*P* value): the probability that their target hexamers are found more often in 3′UTRs of differentially upregulated mRNAs than in unchanged genes by Wilcoxon Rank Sum Test (miR-23b-24-27b family highlighted). (**d**) miR-24 is the only miR-23b-24-27b family member significantly downregulated in aortic tissue at all three different time points during PPE-induced AAA expansion. (**e**) Aortic pri-miR-24 expression at 3 and 7 days after PPE induction of AAA compared with sham. (**f**) ISH for miR-24 (purple chromagen) in untreated (control) aorta, sham and PPE at 14 days after AAA induction (scale bar, 400 μm). (**g**,**h**) Expression of the most significant upregulated (day 7) miR-24 target genes at all tested time points during PPE-induced AAA expansion (versus sham). *n*=5–8 for each group and time point. Data are mean±s.e.m. *P<0.05 versus sham analysed with analysis of variance (ANOVA; one-way repeated measures ANOVA for Fig. 1a) and Bonferroni’s post test.

We profiled miRNA expression in the porcine-pancreatic-elastase (PPE) infusion model in 10-week-old male C57BL/6J mice. The incidence, growth rate and size of aneurysmal expansion were measured by ultrasound (US) at 3, 7, 14, 21 and 28 days after PPE infusion compared with sham (saline-infused) mice (Fig. 1a; Supplementary Table 1 and Supplementary Fig. 1A,B). PPE-induced AAA size differed from sham by day 7. Therefore, we harvested day 7 infrarenal aortic tissue for gene and miRNA microarrays.

When comparing PPE-treated AAA with sham, 41 miRNAs were upregulated with aneurysm and 37 were downregulated (>1.5-fold; *P*<0.05, moderated t-testing with Benjamini-Hochberg FDR correction) (Supplementary Fig. 1C and Fig. 1b). These included miRNAs previously linked to vasculo-pathology, such as miR-21 (increased with aneurysm) and members of the miR-29 family (decreased)^8^,^9^,^10^.

Gene expression analysis identified numerous up- (2,775) and downregulated (2,296) genes at false discovery rate (FDR; <1%) in PPE-treated aortic tissue (versus sham). We cross-correlated miRNA and mRNA expression changes with aneurysm development, identifying which differentially regulated miRNAs showed the most negative correlation with predicted target-gene expression (Targetscan.org), implying biological relevance.

The miR-23b-24-27b cluster had the largest number of negatively correlated targets (213) of any differentially regulated miRNA or miRNA cluster (Fig. 1b; and Supplementary Figs 1D and 2A). Of these genes, 63 were predicted targets of miR-24 alone (Supplementary Fig. 1D). Each cluster member was individually downregulated with AAA. We analysed the 3′-untranslated regions (UTRs) of all significant upregulated AAA genes for miRNA-target seed-hexamer enrichment using DIANA-mirExTra. Of all the downregulated individual miRNAs, miR-24 had the most significant negative correlation with upregulated genes (Fig. 1c)^11^.

The miR-23-27-24 gene cluster occupies two genomic loci in mice and humans. One is intronic (mouse-chr13; human-chr9: miR-23b, miR-27b and miR-24-1) and the second is intergenic (mouse-chr8; human-chr19: miR-23a, miR-27a and miR-24-2)^12^. Mature miR-24 sequences are indistinguishable by quantitative reverse transcription PCR (qRT–PCR). Pri-miR-23b, -27b and -24-1 may be transcribed independently from the cluster gene in mice, although pre-miR-23b may be co-transcribed with pre-miR-27b and pre-miR-24-1 (ref. 13). We measured miR-23b-24-27b cluster expression in infrarenal aortic tissue during the AAA time course. miR-24 remained significantly downregulated at three time points (days 7, 14 and 28; Fig. 1d), while miR-23b and miR-27b were downregulated only at day 7, supporting independent release of individual miRNAs from the cluster-transcript. Further, qRT–PCR from days 3 and 7 showed that pri-miR-24-1 (not pri-miR-24-2) was substantially decreased in aneurysmal tissue (versus sham; Fig. 1e).

*In situ* hybridization (ISH) showed diminished miR-24 expression throughout the aneurysmal aortic wall of PPE mice (versus sham and untreated controls; Fig. 1f).

### miR-24 target-genes in AAA models

We examined the expression of the eight most significantly upregulated miR-24 target mRNAs (from microarray) at baseline and three different time points during PPE-induced AAA development. *Chi3l1*, which belongs to the chitinase-like protein family and is a potential chronic inflammatory disease biomarker^14^, was the only top miR-24 target gene substantially altered at all time points, and showing a complimentary negatively correlated trend versus miR-24 (Fig. 1g,h). We also measured the expression of seven previously validated miR-24 target genes^15^,^16^ (Supplementary Fig. 2B,C). All were upregulated exclusively at day 7, leaving *Chi3l1* as the most compelling miR-24 target during murine AAA development.

**Figure 2:**
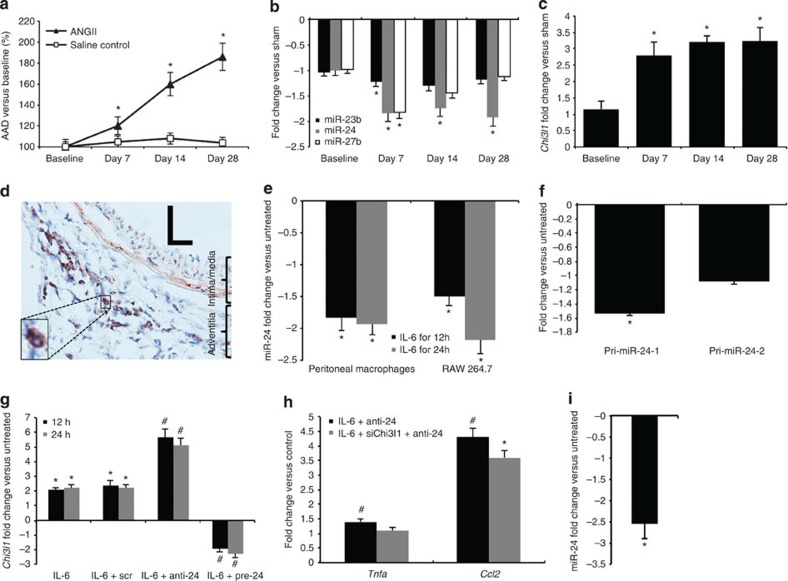
miR-24 expression and downstream effects in angiotensin II-induced AAAs and ***in vitro***. (**a**) Abdominal aortic diameter (AAD; in % versus baseline) expansion in angiotensin II (ANGII)-induced AAA compared with saline-infused control mice. (**b**) miR-23b-24-27b supra-renal aortic tissue expression in ANGII compared with sham (saline control). (**c**) Aortic *Chi3l1* expression in ANGII compared with sham. (**d**) miR-24 (purple chromagen) is co-localized with Chi3l1 protein (brown) in PPE-induced AAA, 7 days after AAA induction (‘L’ indicates the luminal side). High-magnification inset shows co-stained macrophage. (**e**) miR-24 expression in peritoneal macrophages and RAW 264.7 cells, stimulated with interleukin-6 (IL-6) for either 12 or 24 h (versus untreated). (**f**) pri-miR-24 expression in IL-6-stimulated RAW 264.7 cells (24 h). (**g**) *Chi3l1* expression in IL-6-stimulated and miR-24-modulated RAW 264.7 cells (at 12 and 24 h). (**h**) Cytokine gene fold-change in IL-6-stimulated and anti-miR-24±si*Chi3l1*-transfected RAW 264.7 s (24 h). (**i**) miR-24 expression in IL-6-stimulated human aortic SMCs (24 h). *n*=5–9 for each group and time point. Data are mean±s.e.m. *P<0.05 versus sham (saline controls). ^*#*^*P*<0.05 versus untreated/control and other treatment groups analysed with analysis of variance (ANOVA; one-way repeated measures ANOVA) and Bonferroni’s post test or Student’s *t*-test (two-tailed; Fig. 2i).

We confirmed the above results in another AAA model, systemically infusing angiotensin II (ANGII) into 10-week-old male *ApoE*−/− mice over 28 days. Compared with saline-infused controls, the suprarenal abdominal aortic diameter (AAD) significantly increased with ANGII by day 7 (Fig. 2a and Supplementary Table 2). As expected for this model, the mortality rate due to aortic rupture and dissection was significantly higher in ANGII mice (28%) versus saline controls (0%). Again, miR-24 was the only member of the miR-23b-24-27b cluster to be significantly downregulated at all three time points (days 7, 14 and 28) during aneurysm development (Fig. 2b). *Chi3l1* expression was again negatively correlated (increased) with miR-24 expression (Fig. 2c). As expected and previously reported by others^17^, ANGII treatment raised blood pressure values significantly. No blood pressure alteration was detectable with PPE-induced AAA induction (Supplementary Table 3).

### miR-24 and its target Chi3l1 regulate inflammation

ISH for miR-24 and immunohistochemistry (IHC) using anti-F4/80 (a macrophage inflammatory marker) revealed that miR-24 co-localized with activated macrophages in aneurysmal aortic mouse tissue (post-PPE day 7; Fig. 2d). miR-24 expression was also visualized in the aneurysm intimal–medial region. Double immunofluorescence of the vascular SMC-marker α-actin (SMA) and F4/80 with Chi3l1 indicated co-localization (orange in merged images) of Chi3l1 with both SMCs and macrophages (Supplementary Fig. 2D).

We explored the regulatory role of miR-24 on inflammation and CHI3L1 expression in HEK293 and aneurysm-related cell types *in vitro*. First, via HEK293 transfection, direct suppression of CHI3L1 transcription through 3′UTR binding of miR-24 was confirmed by luciferase assay (Supplementary Fig. 2E). Next, peritoneal macrophages obtained from ANGII–AAA mice and untreated mice were stimulated with recombinant interleukin (IL)-6 to induce inflammation. Parallel experiments utilized the murine macrophage cell line, RAW 264.7. IL-6 treatment decreased miR-24 expression compared with control cells at two time points (Fig. 2e). As observed *in vivo,* decreases in macrophage miR-24 with IL-6 stimulation were due to reductions in pri-miR-24-1 (Fig. 2f). Further, IL-6 treatment increased expression of *Chi3l1* (Fig. 2g).

Macrophage miR-24 expression was modulated through transfection with either an antagomiR (anti-24) to inhibit or a pre-miR (pre-24) to overexpress miR-24 (versus scrambled-miR control; scr-miR). In both macrophage lines, anti-24 augmented the IL-6-induced *Chi3l1* increase, whereas pre-24 countered IL-6, driving *Chi3l1* expression below scr-miR-treated baseline, further confirming miR-24 regulation (Fig. 2g and Supplementary Fig. 3A). miR-24 downregulation was pro-inflammatory in macrophages, augmenting expression of mediators Tnf-α and Ccl2/Mcp-1 (Fig. 2h). This process involved Chi3l1, as simultaneous >75% short interfering RNA (siRNA) knockdown (siChi3l1) reduced anti-24-induced increases in inflammatory gene expression (Fig. 2h). These results suggest that IL-6 (abundant in developing AAA) decreases miR-24-1 expression in macrophages, leading to a rise in Chi3l1. This combination augments macrophage activation, increasing inflammatory cytokines.

**Figure 3:**
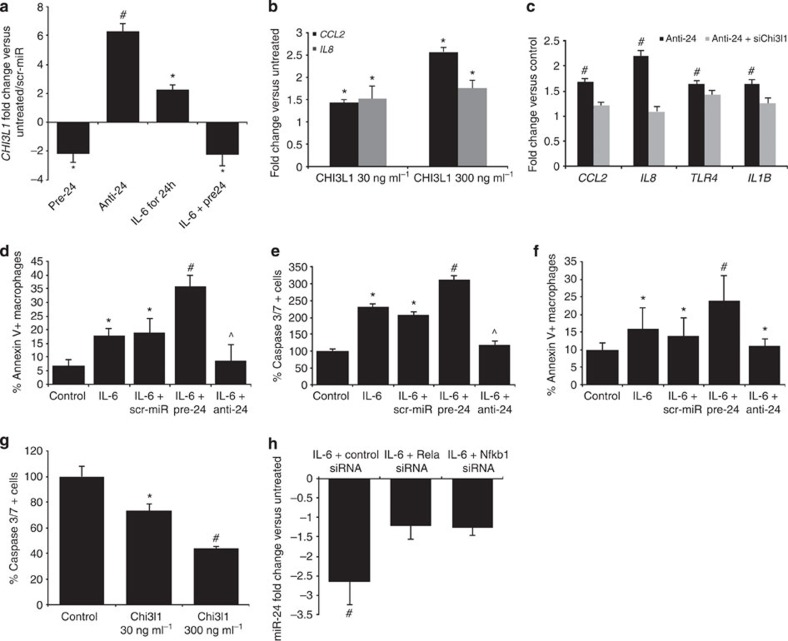
Regulation and modulation of miR-24 *in vitro*. (**a**) *CHI3L1* expression in miR-24-modulated and IL-6-stimulated human aortic SMCs. (**b**) Cytokine expression in CHI3L1-treated human aortic SMC. Data are mean±s.e.m. (**c**) Cytokine expression in IL-6-stimulated, miR-24-modulated and si*Chi3l1*-transfected hASMC. (**d**) Apoptosis in IL-6-stimulated and miR-24-modulated peritoneal mouse macrophages from mice with ANGII-induced AAA. (**e**) Apoptosis in IL-6-stimulated and miR-24-modulated RAW 264.7 cells. (**f**) Apoptosis in IL-6-stimulated and miR-24-modulated peritoneal mouse macrophages from mice without ANGII-induced AAA. (**g**) Percent apoptosis in Chi3l1-treated RAW 264.7 cells. (**h**) Inhibition of NF-κB via silencing of its components (*RelA* or *Nfkb1*) prevents miR-24 downregulation in IL-6-stimulated peritoneal macrophages. *n*=3–4 for each treatment and cell type; all experiments were repeated three times. Data are mean±s.e.m. *P<0.05 versus control, untreated or untreated/scr-miR. ^*#*^*P*<0.05 versus untreated/control and other treatment groups. ^*P*<0.05 versus sham, scr-miR and anti-24, analysed with analysis of variance and Bonferroni’s post test.

These same cellular responses were also observed in cultured primary human aortic SMC. IL-6 stimulation decreased miR-24 (Fig. 2i) and increased *CHI3L1* expression (Fig. 3a). As in macrophages, miR-24 modulation in SMCs altered *CHI3L1* expression (Fig. 3a). SMC transfection with anti-24 induced (and pre-24 inhibited) inflammatory gene expression (for example, *CCL2* and *IL-8*; Supplementary Fig. 3B,C). Further, recombinant CHI3L1 treatment of SMCs led to dose-dependent increases in inflammatory gene expression (Fig. 3b). CHI3L1 treatment increased *CCL2* expression, a response augmented by IL-6 (Supplementary Fig. 3D). Again, successful siCHI3L1-mediated knockdown in SMC largely eliminated pro-inflammatory effects of anti-24 (Fig. 3c). Successful miRNA modulator transfection (>80%) was confirmed by fluorescent microscopy and fluorescence-activated cell sorting (FACS) for labelled tag (Supplementary Fig. 3E).

CHI3L1 can induce bronchial SMC migration through JNK and ERK phosphorylation^18^. We found that CHI3L1 dose-dependently increases JNK and ERK phosphorylation in human aortic SMCs (Supplementary Fig. 4A,B). Appropriately, similar effects on JNK and ERK phosphorylation were also observed after miR-24 modulation (Supplementary Fig. 4C,D). Further, treatment with CHI3L1 dose-dependently increased aortic SMC migration *in vitro* (Supplementary Fig. 4E).

We next examined the effects of miR-24 and Chi3l1 on macrophage survival. Overexpression of miR-24 in peritoneal macrophages and RAW 264.7 increased apoptosis (Annexin V+ and caspase 3/7 assays) beyond that seen with IL-6 stimulation alone, while anti-24 abrogated the pro-apoptotic effects of IL-6 (Fig. 3d,e). These effects on macrophage survival were also detectable in peritoneal macrophages derived from mice without ANGII-induced AAA (Fig. 3f). Further, treatment of RAW 264.7 cells with recombinant Chi3l1 dose-dependently attenuated apoptosis (Fig. 3g). miR-24 modulation inversely altered both *Chi3l1* expression and Chi3l1 protein levels in peritoneal macrophages and RAW 264.7 cells. Further, anti-24 increased (while pre-24 decreased) phospho-Akt levels, partly explaining miR-24-based macrophage apoptosis regulation (Supplementary Fig. 5A,B).

The above studies involved primarily M1-subtype pro-inflammatory macrophages. We examined the anti-inflammatory M2-subtype by treating RAW cells and peritoneal macrophages with low (5 ng ml^−1^) and high (30 ng ml^−1^) dose IL-4. The M2-marker macrophage mannose receptor 1 (*Mrc1*) markedly increased with both doses (Supplementary Fig. 5C). Neither *Chi3l1* nor miR-24 expression was significantly altered with IL-4 treatment, although there was a trend towards downregulation of Chi3l1 and upregulation of miR-24 in RAW 264.7 cells (Supplementary Fig. 5D,E). Further experiments on macrophage polarization, utilizing recombinant IL-4 and IL-6 stimulation of murine peritoneal macrophages, revealed that miR-24 modulation predominantly affects M1 prototypical macrophage markers, such as *Il12* and *Nos1* (Supplementary Fig. 5F,G), but not those of the M2 subtype, as no substantial change was observed in the expression of *Il10* and *Arg1* (Supplementary Figs 5H and 6A).

We investigated nuclear factor-κB (NF-κB) as a regulator of miR-24 in IL-6-stimulated RAW cells. The IL-6-induced decrease in miR-24 was essentially eliminated in macrophages by prior siRNA-mediated knockdown (>75%) of either *RelA* (p65) or *Nfkb1* (p50), key components of NF-κB (Fig. 3h).

Next, we examined inflammation in human aortic endothelial cells (AoECs) in response to CHI3L1 treatment, with and without IL-6 co-treatment. CHI3L1 dose-dependently augmented the effects of IL-6 treatment on *CCL2* expression and increased expression of several adhesion molecules (*VCAM1, ICAM1* and *SELP*; Supplementary Fig. 6B–E). These results suggest that elevated levels of CHI3L1 augment inflammatory stimuli, increasing monocyte and white-blood cell binding via increases in adhesion molecules.

Furin, a proprotein convertase, has been shown to be a direct target of miR-24 in cardiac fibroblasts^19^. Because, furin has previously been shown to indirectly regulate matrix metalloproteinases-2 and -9, and to control latent transforming growth factor beta (TGF-β) activation processing (key players in AAA pathobiology)^20^,^21^, we evaluated whether miR-24 modulation *in vitro* would alter furin in human aortic SMCs. Interestingly, pre-24 significantly suppressed furin expression, whereas anti-24 treatment had no significant effect (Supplementary Fig. 6F).

Finally, we investigated differential mechanisms of transcription-dependent processing of the three different miR-23b-24-27b cluster members by performing actinomycin D transcript degradation experiments^22^,^23^ in IL-6-stimulated RAW 264.7 cells. The results indicate that all three miRNAs have about the same slow basal degradation/processing rate (Supplementary Fig. 6G). Interestingly, IL-6 markedly increases miR-24 degradation/processing, an effect that appears to be transcription-dependent (Supplementary Fig. 6H). Further, IL-6 has a minor effect on miR-23b levels at 24 h (Supplementary Fig. 7A). It also causes a transient minor increase in miR-27b expression (Supplementary Fig. 7B).

### Pre-miR-24 inhibits AAA development in mouse models

**Figure 4:**
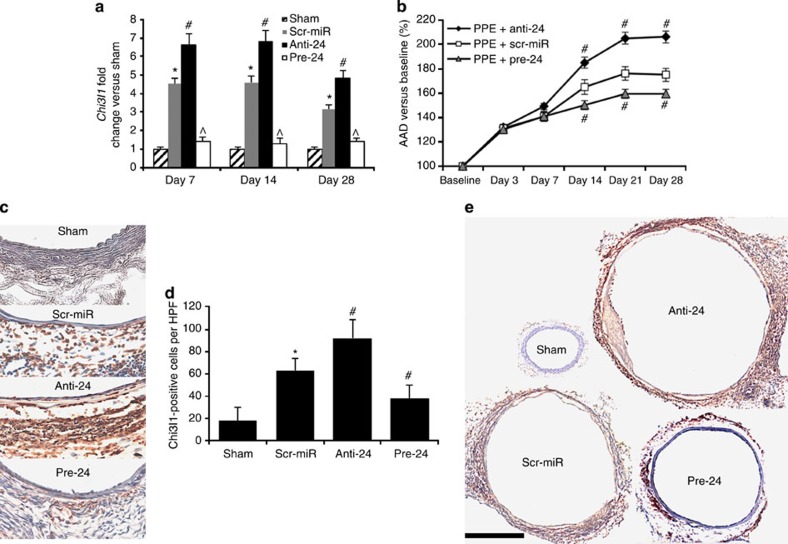
miR-24 modulation *in vivo*. (**a**) *Chi3l1* aortic expression at different time points in scr-miR and anti-/pre-24-transduced mice in sham or PPE-induced AAA at various time points. (**b**) Abdominal aortic diameter (AAD; in % versus baseline) time course in scr-miR and anti-/pre-24 PPE-induced AAA. (**c**) IHC for aortic Chi3l1 (brown chromagen) in anti-/pre-24-treated mice compared with scr-miR, 14 days after PPE induction of AAA. (**d**) Chi3l1-positive cell counts (*n*=4 high-power fields (HPFs) on three different sections of three different aortas per group) in miR-24-modulated mice (versus scr-miR control), 14 days after PPE–AAA induction. (**e**) Representative IHC images (antibody against murine Chi3l1) in different-sized aneurysms of miR-24-modulated mice (pre-/anti-24) compared with sham and scr-miR (scale bar, 50 μm), 28 days after PPE–AAA induction. Data are mean±s.e.m. *P<0.05 versus sham. ^#^*P*<0.05 versus scr-miR and sham. ^*P*<0.05 versus sham, scr-miR and anti-24; analysed with analysis of variance (ANOVA; one-way repeated measures ANOVA for Fig. 4b and Bonferroni’s post test.

We investigated whether modulation of miR-24 affects murine PPE–AAA development, expansion and rupture, utilizing lentiviral vector (7.6 × 10^7^ IFU per ml) transfection of pre-miR-24 (pre-24), anti-miR-24 (anti-24) or scr-miR. Successful miR-24 inhibition and overexpression *in vivo* were confirmed by qRT–PCR (Supplementary Fig. 7C). Pre-24 significantly decreased *Chi3l1* expression at all time points (days 7, 14 and 28) compared with scr-miR in aneurysmal tissue, while anti-24 transduction in PPE mice led to increased *Chi3l1* at all three time points versus the other two treatments (Fig. 4a). Modulation of miR-24 expression did not affect blood pressure in transduced mice (Supplementary Table 6).

Overexpressing miR-24 substantially attenuated increases in AAD, while further inhibition with anti-24 augmented AAD expansion (Fig. 4b and Supplementary Table 4). Two anti-24 mice died of AAA rupture (at days 11 and 24), uncommon for the PPE infusion model. miR-24 modulation had minimal impact on *Chi3l1* in the suprarenal (non-aneurysmal) abdominal aorta, suggesting uptake of miRNA modulators only at the site of injury (Supplementary Fig. 7D).

**Figure 5:**
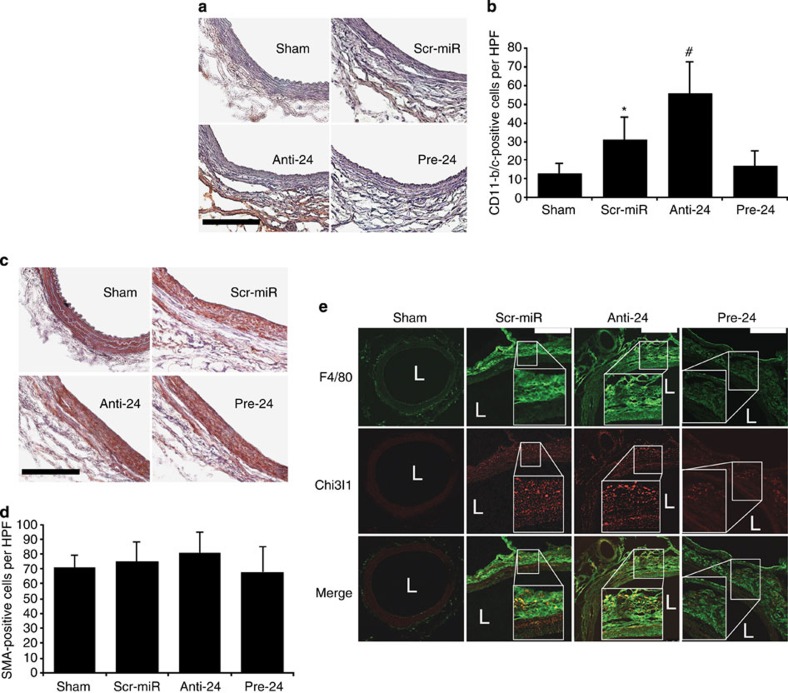
Effects of miR-24 modulation *in vivo*. (**a**) IHC for CD11b/c in anti-/pre-24-treated mice compared with scr-miR and sham, 14 days after PPE induction of AAA (brown chromagen; scale bar, 400 μm). (**b**) CD11b/c-positive cell counts (*n*=4 high-power fields on three different sections of three different aortas per group) in miR-24-modulated mice, 14 days after PPE–AAA induction (versus scr-miR and sham). (**c**) IHC for SMC α-actin (SMA) in anti-/pre-24-treated mice compared with scr-miR and sham, 14 days after PPE induction of AAA (brown chromagen; scale bar, 400 μm). (**d**) SMA-positive cells (*n*=4 high-power fields on three different sections of three different aortas per group) in miR-24-modulated mice, 14 days after PPE–AAA induction (versus scr-miR and sham). (**e**) Confocal microscopy illustrating co-localization and decreased levels of F4/80 and Chi3l1 in pre-24-transfected mice with PPE–AAA (at 14 days) compared with sham (scale bar, 50 μm), scr-miR- and anti-24-transfected mice (‘L’ indicates the luminal side; scale bars, 400 μm). *n*=4–6 mice for each group. Data are mean±s.e.m. ^*#*^*P*<0.05 versus scr-miR and sham. *P<0.05 versus sham and anti-24.

IHC staining for Chi3l1 confirmed decreased protein levels throughout the vessel wall in pre-24-transfected mice compared with scr-miR and sham (Fig. 4c–e). Aortic Chi3l1 protein levels rose in anti-24 mice (Fig. 4d). Macrophage infiltration was significantly decreased in pre-24-treated mice (Fig. 5a,b,e and Supplementary Fig. 7E). SMA was minimally altered in anti-24 mice (versus scr-miR; Fig. 5c,d; whole aortas are displayed in Supplementary Fig. 7F). Confocal microscopy of double-immunofluorescence-stained aortas with anti-F4/80 and -Chi3l1 confirmed altered levels of both with miR-24 modulation and co-localization within PPE-induced AAAs (Fig. 5e).

**Figure 6:**
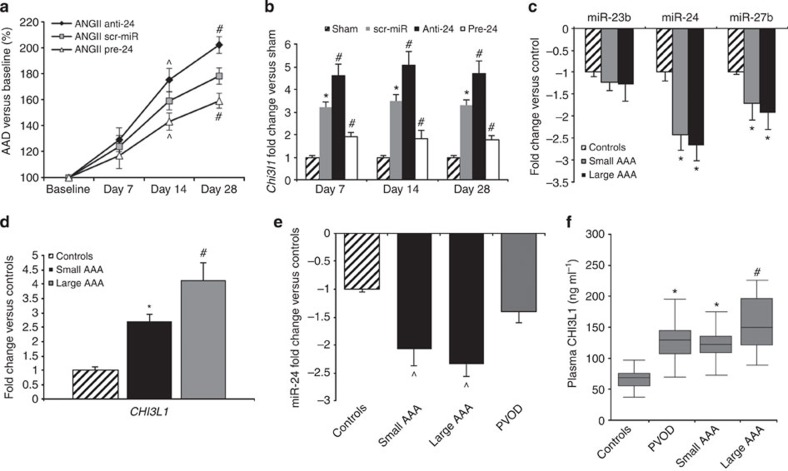
miR-24 modulation in ANGII-induced AAA and levels in human AAA. (**a**) Abdominal aortic diameter (AAD; in % versus baseline) in scr-miR and anti-/pre-24 ANGII-induced AAA (*n*=4–6 mice for each time point and group). (**b**) *Chi3l1* aortic expression in scr-miR- and anti-/pre-24-transfected mice compared with sham in the ANGII–AAA model at various time points. (**c**) miR-23b-24-27b expression in human aortic tissue samples from patients with small AAA (*n*=12) and large AAA (*n*=10), compared with tissue from control patients without AAA (*n*=14). (**d**) *CHI3L1* is significantly upregulated in human tissue samples with large AAA compared with small AAA and non-aneurysmal aortic tissue. (**e**) miR-24 is significantly downregulated in plasma samples from human patients with AAA (*n*=43 with small AAA; *n*= 39 with large AAA) compared with control plasma (*n*=44), and patients with peripheral vascular occlusive disease (PVOD) but no AAA (*n*=22). (**f**) CHI3L1 plasma levels are significantly increased in patients with large AAA compared with patients with small AAA, PVOD and un-diseased controls. Data are mean±s.e.m. *P<0.05 versus sham/controls. ^*#*^*P*<0.05 versus scr-miR/controls/small AAA. ^*P*<0.05 versus pre-/anti-24 or versus controls/PVOD; analysed with analysis of variance (ANOVA; one-way repeated measures ANOVA for Fig. 6a) and Bonferroni’s post test.

Pre-24 treatment also attenuated AAA in the ANGII model, although the results were less pronounced than in the PPE model. Significant changes in AAD could be detected by 14 days (Fig. 6a and Supplementary Table 5). *Chi3l1* was again significantly downregulated in pre-24-treated versus the other two groups (Fig. 6b). Death from aortic rupture occurred significantly more often in anti-24-treated mice (58%), compared with scr-miR- (36%) and pre-24-transfected (12%) through day 28. miR-24 expression in anti-/pre-24-transduced mice was less altered than in the PPE model (Supplementary Fig. 8A). We have described this phenomenon previously^8^,^9^ and attribute it to improved miRNA modulator uptake at the PPE injury site, while the more intact aortic wall found in the ANGII model may decrease anti-/pre-miR uptake. Notably, when analysing *Chi3l1* expression within the infrarenal aorta after ANGII induction, we detected slight alteration of gene transcription at 14 days (Supplementary Fig. 8B). This may well relate to the minor AAD increase that occurs in this area after ANGII infusion.

### miR-24 modulation effects on gene expression in murine AAA

We collected infrarenal aortic tissue from miRNA-modulated and sham-controlled mice 7 days after PPE infusion, and hybridized isolated RNA to whole-genome microarrays. Ontologic pathway enrichment of significant genes was performed as previously described^24^. In brief, numerous genes differentiated scr-miR-treated PPE-induced AAA from scr-miR-treated sham-control aorta. In contrast, anti-24-modulated PPE-infused gene expression was essentially indistinguishable from scr-miR-treated PPE-infused mice. *Chi3l1* remained upregulated with anti-24 treatment. Gene expression in pre-24-modulated aorta was similar overall to sham controls, including undetectable *Chi3l1* expression (Supplementary Table 7 and Supplementary Note 1).

### miR-24 expression and Chi3l1 in human AAA

Human infrarenal aortic tissue was obtained from patients (*n*=22) undergoing elective open surgery for significantly enlarged abdominal aortae (AAD: 52–115 mm). We compared this group of AAA patients (aged 68±13 years) with infrarenal aortic tissue from a group of control organ donors without AAA (*n*=14, mean age 34±16 years) at explant (*n*=10 heart; *n*=4 kidney; Fig. 6c,d). All AAA patients were on similar medications at time of surgery, and were male, Caucasian, non-diabetic and smokers.

As in our animal models, miR-24 was significantly downregulated (−1.9±0.09-fold) in human AAA tissue (versus control). miR-27b was also downregulated in AAA (Fig. 6c). There were no differences in miR-24 levels between patients with small (52–67 mm; *n*=12) and large AAA (69–115 mm, *n*=10), although there was a trend towards lower miR-24 with larger AAA. Tissue expression of *CHI3L1* positively correlated with disease severity (Fig. 6d).

### miR-24 and CHI3L1 are novel AAA biomarkers

We isolated plasma RNA from 105 individuals at the time of surgical AAA repair, and compared them with a group of 52 age-, medication- and risk-factor-matched controls with normal renal/liver function (Supplementary Table 8), and to a separate 22 patient group with isolated peripheral vascular occlusive disease (PVOD). All individuals had no documented current or previous other cardiovascular event (for example, stroke and myocardial infarction).

We detected decreased plasma miR-24 expression in patients with small (45–67 mm; *n*=54) and large AAA (69–150 mm; *n*=51; Fig. 6e) compared with both controls and PVOD patients. As in aortic tissue, miR-24 plasma levels were indistinguishable between patients with small or large AAAs. In contrast, there were significant differences in CHI3L1 plasma levels between patients with small and large AAA, and between AAA patients and controls (Fig. 6f). CHI3L1 levels in subjects with small AAA were comparable to those with PVOD, but levels in patients with large AAA were significantly higher than in both of the other two groups.

We also investigated changes in circulating miR-24 plasma expression and Chi3l1 protein levels in our two murine AAA models, and discovered that plasma miR-24 was significantly repressed in mice with aneurysms (Supplementary Fig. 8C). A substantial additional decrease was observed in mice with dissected/ruptured AAAs from the ANGII model. Chi3l1 levels were consistently elevated with AAA expansion in both models and even further with subsequent rupture in the ANGII model (Supplementary Fig. 8D).

## Discussion

miRNAs offer translational opportunities for innovative therapeutic and biomarker applications. Signature miRNA (dys)regulation patterns exist for several cardiovascular disorders, including cardiac hypertrophy^25^, myocardial infarction^26^, atherosclerosis^27^ and peripheral artery disease^28^. Further, murine gain- and loss-of-function studies have demonstrated the crucial importance of specific miRNAs in orchestrating pathophysiologic processes.

The highly conserved miR-23b-24-27b cluster is involved in post-infarct cardiac angiogenesis^15^, cardiomyocyte survival^29^ and cancer^30^,^31^. We now report that miR-24 is a key regulator of aortic inflammation in AAA, with pri-miR-24-1 being the relevant transcript (miR-24-2 is predominant in tumour pathologies^32^,^33^,^34^).

CHI3L1 (a 18-glycosyl hydrolase family member)^14^ is a predicted target of miR-24 involved in acute and chronic inflammation^35^,^36^. Differentiated macrophages secrete CHI3L1 in early-stage atherosclerotic lesions^37^,^38^, while CHI3L1 analogues induce vascular smooth muscle cell (VSMC) proliferation and migration^39^. Elevated CHI3L1 serum levels are found in lung disease^40^ and have predictive value in detecting elevated future risk for thromboembolic stroke in healthy women^41^. CHI3L1 increased IL-8 production in bronchial SMCs via mitogen-activated protein kinase (MAPK) and NF-κB pathways, triggering inflammation and SMC migration^18^. These data suggest a vascular disease role for CHI3L1.

*In vivo* (mouse), *in vitro* (cell culture) and *ex vivo* (human tissue and plasma), we identified miR-24 as a key regulator of AAA initiation and propagation, which acts in part by targeting CHI3L1 (Supplementary Fig. 9). Analysis suggested *Chi3l1* as an intriguing miR-24 target. miR-24-*Chi3l1* interactions controlled inflammatory activity within the dilated aortic wall, effects abrogated by pre-miR-24 transfection. miR-24 overexpression blocked *Chi3l1* induction *in vivo*, limiting AAA expansion, while anti-miR-24 led to higher *Chi3l1* expression and development of larger, rupture-prone aneurysms.

Microarray studies examining miR-24-modulated aortic tissue confirmed that pre-miR-24 transfection led to AAA-associated-pathway reductions, including immune response and cytokine activity. Anti-miR-24 transfection primarily augmented the degree of inflammatory and apoptosis-related responses rather than activating/repressing alternative pathways, suggesting that the observed miR-24 downregulation with aneurysm is pathologic rather than homeostatic. This stands in contrast to miR-29b, for which further *in vivo* repression beyond that seen with AAA development led to fibrosis and decreased aneurysm size^9^.

We explored potential mechanisms behind the pathology. Inflammatory stimuli downregulated miR-24 in macrophages and vascular SMCs at least partly via NF-κB. While NF-κB is widely established as an activator of transcription, it may also act as a transcriptional repressor^42^,^43^,^44^. miR-24 directly modulated *CHI3L1* expression levels and, through CHI3L1, controlled cytokine synthesis, altered cell survival (probably through p-Akt) in macrophage cell lines, promoted cytokine production and migration in aortic SMCs, and stimulated adhesion molecule expression in AoECs.

As mentioned above, CHI3L1 treatment activates MAPK pathways in bronchial SMCs^18^. MAPK signalling appears critical in AAA, as selective JNK inhibition in rodent models prevents aneurysm development^45^, while ERK1(−/−) mice are protected from PPE-induced AAA^46^. We show that both CHI3L1 treatment and miR-24 inhibition activate JNK/ERK in SMC *in vitro,* while pre-miR-24 decreases phospho-JNK/ERK, suggesting additional mechanistic links between miR-24/CHI3L1 regulation and AAA.

CHI3L1 appears to be a crucial regulator of transmural vascular inflammation in AAA and its regulation by miR-24 suggests potential therapeutic approaches. However, as miRNAs may promote disease in one tissue while being protective elsewhere, safe miRNA modulator application will ultimately require methods that minimize off-target effects. We utilized human immunodeficiency virus type 1-derived lentivirus to modulate miR-24 during murine AAA development. These lentiviruses lack the U3 promoter, leading to self-inactivation^47^. For vascular diseases such as AAA, the use of local delivery devices such as miRNA-modulator-eluting stents/balloons could resolve off-target issues, increasing therapeutic feasibility. No other direct antagonistic targeting agent for CHI3L1 exists at this time, but future availability of such reagents will enable deeper investigation as to the role, function and therapeutic potential of targeting CHI3L1 in cardiovascular disease.

To date, biomarkers have failed to improve diagnostic and prognostic precision in AAA. Recent data show that CHI3L1 is elevated in patients with coronary artery disease, and that disease progression corresponds with augmented CHI3L1 levels^48^. We found that AAA size also correlates with higher CHI3L1 levels. Circulating miRNAs also offer promise as biomarkers in that they are stable in human blood, detectable with high sensitivity/specificity and measurable via miRNA microarrays and qRT–PCR arrays^49^,^50^. Cardiovascular studies have identified miRNA dysregulation in various specimens, although typically underpowered and/or without matched controls^43^,^44^,^45^,^46^,^47^,^48^,^49^,^50^,^51^. In our study, plasma and tissue levels of miR-24 were significantly decreased in AAA patients, but unlike CHI3L1 these were not clear disease-severity indicators. Larger patient cohorts are needed to validate miR-24 or CHI3L1 as diagnostic biomarkers for AAA. More intriguing, miR-24 could perhaps be used prospectively to detect patients at high risk for rapid AAA growth/rupture. Grouped miRNAs might show increased predictive power over single transcripts, as recently reported for myocardial infarction^52^. Notably, miR-24 was one of the circulating miRNAs biomarkers in the cited study, suggesting that it might also detect patients at increased risk of future myocardial infarction. However, a combination of both CHI3L1 and miR-24 could potentially be utilized in patients for AAA detection and rupture risk stratification.

## Methods

### PPE infusion model

To induce murine AAA, the PPE infusion model was performed in 10-week-old male C57BL/6J mice^53^. In brief, after placing temporary ligatures around the proximal and distal aorta, an aortotomy was created at the bifurcation and an insertion catheter was used to infuse the aorta for 5 min at 100 mm Hg with saline or saline-containing type I PPE (1.5 U ml^−1^; Sigma-Aldrich). After removing the infusion catheter, the aortotomy was repaired without constriction of the lumen. Two aortic segments were harvested at 7, 14 or 28 days post surgery: the induced AAA (area between the left renal artery and the bifurcation) and the suprarenal abdominal aorta (area proximal to the renal arteries, up to the diaphragm) were snap-frozen in liquid nitrogen and then stored at −80 °C pending further processing.

### ANGII infusion model

Osmotic pumps (model 2004, Alzet) containing either ANGII (1 μg kg^−1^ min^−1^, Sigma-Aldrich) or saline were implanted in 10-week-old *ApoE*−/− male mice (C57BL/6J background)^54^. Aneurysmal (proximal to the renal arteries) and control segments (distal to the renal arteries) of the aorta were harvested after 14 or 28 days, snap-frozen in liquid nitrogen and then stored at −80 °C pending further processing.

### Aortic diameter measurements by US imaging

At baseline, and 3, 7, 14, 21 and 28 days after aneurysm induction, B-mode US imaging was performed to assess the AAD. Mice were anaesthetized using 2% isoflurane (Vet One) and were laid supine on a heated 37 °C plate. Two-dimensional B-mode imaging was performed using a real-time microvisualization scan head (RMV 704) with a central frequency of 40 MHz, frame rate of 30 Hz, a focal length of 6 mm and a 20 × 20 mm field of view (VisualSonics). Transverse image scans were performed and cine loops of 300 frames were acquired throughout the infra- and suprarenal region of the mouse aorta. The acquired images were stored digitally on a built-in hard drive for offline analysis to determine maximal AAD. All aortic diameters were measured in anterior–posterior direction during the diastolic phase. US image analysis was performed using the accompanying Vevo770 software (VisualSonics). Measurements were accomplished using random selection of each data set and operator blinding to prevent recall bias. All measurements were collected by one observer to limit bias, while results were analysed by a second independent observer blinded to the treatment group.

### Preparation of aortic tissue

Mice were killed with an inhalation overdose of isoflurane (Vet One). Immediately afterward, the abdominal aorta was transected and flushed via the left ventricle with ice-cold PBS. The aorta was then dissected from fat and connective tissue from the left renal artery to the bifurcation under a microscope (Leica). Specimens were snap-frozen individually in liquid nitrogen and stored at −80 °C before further processing.

### RNA quantification

Total RNA was isolated using a TRIzol-based (Invitrogen) RNA isolation protocol. RNA was quantified by NanoDrop (Agilent Technologies), and RNA and miRNA quality were verified using an Agilent 2100 Bioanalyzer (Agilent Technologies). Samples required 260/280 ratios >1.8, and sample RNA integrity numbers ≥9 for inclusion. RNA was reverse transcribed using the TaqMan microRNA Reverse Transcription kit (Applied Biosystems) according to the manufacturer’s instructions. MiRNA and TaqMan assay kits (Applied Biosystems) for miR-23b (5′-AUCACAUUGCCAGGGAUUACC-3′), miR-24 (5′-UGGCUCAGUUCAGCAGGAACAG-3′), miR-27b (5′-UUCACAGUGGCUAAGUUCUGC-3′), sno202 (endogenous control for normalization in mice) and RNU44 (control for human samples) were used. For mRNA, the iScript cDNA synthesis kit (Bio-Rad) was used to synthesize first-strand cDNA according to the manufacturer’s protocol. TaqMan qRT–PCR assays were performed using mouse- and human-specific primers (Applied Biosystems). Relative expression of miR-24-1 and miR-24-2 clusters were obtained by TaqMan for pri-miR-24-1 (5′-CUCCGGUGCCUACUGAGC UGAUAUCAGUUCUCAUUUUACACACUGGCUCAGUUCAGCAGGAACAGGAG-3′) and pri-miR-24-2 (5′-GCCUCUCUCCGGGCUCCGCCUCCCGUGCCUACUGAGCUGAAACAG UUGAUUCCAGUGCACUGGCUCAGUUCAGCAGGAACAGGAGUCCAGCCCCCUAGGAGCUGGCA-3′; both from Applied Biosystems). All non-miRNA probes were normalized to 18S as a multiplexed internal control. Amplification took place on either a PRISM 7900HT or a QuantStudio12K Flex (Applied Biosystems). All fold changes were calculated by the method of ΔΔ*C*_t_, and are expressed as mean±s.e.m. compared with either sham-operated mice, saline-infused mice, saline-treated cells (*in vitro*) or human control patients (as indicated). The amount of samples per experiment is indicated in the corresponding figure legend and/or the results.

### Gene microarray hybridization

Un-pooled aortic RNA (500 ng) isolated as described was labelled using Cyanine-5-CTP and hybridized to the SurePrint G3 Mouse Whole Genome GE 8 × 60 K Microarray G4852A platform with an equimolar concentration of Cyanine-3-CTP-labelled universal mouse reference (Stratagene). These same aortic samples (100 ng total RNA) were also labelled with Cyanine-3-CTP and hybridized to the Agilent Mouse miRNA Microarray, 8 × 15 K G4471A-021828 platform. Standard protocols were followed, with one mouse aorta per array. Images were quantified and feature-extracted using Agilent Feature Extraction Software (version A.10.7.3.1). All arrays were of the same print run and were hybridized on the same day.

### Microarray quality control and data analysis

A small number of arrays were excluded from further analysis for poor hybridization characteristics per feature extractor quality control metrics. Remaining array data were examined using Genespring 12.6.1 (Agilent Technologies). Hierarchical analysis clustering and principal components analysis was used to confirm and identify appropriate treatment subgroups and eliminate outliers. Final analysis groups included two different sets consisting of 7-day PPE-treated versus sham-saline-treated aortae (five arrays each) and 7-day PPE-treated-miR-24-modulated aortae versus controls (sham-operated saline-treated (four arrays), scr-miR-PPE-treated (five arrays), pre-miR-24-PPE-treated (three arrays) and anti-miR-24-PPE-treated (six arrays)). For gene expression data, two-class, unpaired significance analysis of microarrays (SAM v.4.0) was performed in every combination with FDR <1% (ref. 55). miRNA array data were analysed in Genespring, using moderated *t*-testing with Benjamini–Hochberg FDR correction (*P*<0.05). Gene lists were probed for overabundant (two genes per category minimum, EASE cutoff 0.05) pathways (Kyoto Encyclopedia of Genes and Genomes) and Gene Ontology Biological Process groups using DAVID Bioinformatics Resources 6.7 against a murine background^56^.

Predicted target 3′UTR seed region hexamers were identified for all differentially regulated miRNAs (*P*<0.05 PPE versus saline, Wilcoxon Rank Sum), and the differentially regulated mRNA genes were probed for enrichment of these seed regions using DIANA-mirExTra. Returned values show the probability that hexamers are found more often in the differentially regulated list than in the non-regulated list, as given by Wilcoxon Rank Sum Test^11^. Microarray data were submitted to the National Center for Biotechnology Information's Gene Expression Omnibus (GSE51229).

### Histological and immunohistochemical analysis

Histological staining after harvesting was performed in the same region of abdominal aorta that was imaged in order to obtain morphometric data to correlate with US measurements and gene expression results from qRT–PCR using a standardized protocol^57^. Aortic tissue was divided into four (spaced 0.5 mm apart) 7-μm-thick serial sections from the left renal artery to the bifurcation and stained with haematoxylin and eosin (Sigma-Aldrich), and with rabbit antibodies against Chi3l1, SMA, F4/80 and CD11b/c (all from Abcam, dilution 1:200) using the Vectastain ABC kit (Vector Laboratories) for immunohistochemical analysis. For negative control, mouse irrelevant IgG1 (Abcam) was utilized. Chi3l1-, SMA- and CD11b/c-positive cells were measured by counting the cells in four high-power fields on three different sections of three different aortas per treatment group. Counting was performed by a blinded investigator, 7 days after PPE infusion to induce AAA. All histological analyses were obtained at room temperature using a Zeiss Axioplan 2 (Carl Zeiss MicroImaging) with Zeiss Achroplan and Zeiss Plan-Neofluar lenses, a Nikon Digital Sight (DS) Ri1 camera and the NIS-Elements F 3.00 software (Nikon).

### *In situ* hybridization

ISH for miR-24 was performed by using the miRCURY LNA microRNA ISH Optimization Kit (Exiqon), and 5′-DIG- and 3′-DIG-labelled probes for mmu-miR-24 according to the manufacturer’s protocol. The sequence of the LNA miR-24 control probe was: 5′-DIG/CTGTTCCTGCTGAACTGAGCCA/DIG-3′.

### Combined ISH/immunohistochemical staining

ISH was performed as described above utilizing the miRCURY LNA microRNA ISH Optimization Kit (Exiqon) and 5′-DIG- and 3′-DIG-labelled probes for mmu-miR-24. After finishing the ISH procedure, the sample slides were incubated with pepsin in HCl (1.3 mg ml^−1^ with an adjusted pH between 1.3 and 1.5) for 8 min at 37 °C. After washing in PBS (twice for 5 min), the immunohistochemical staining (as described above) was initiated with overnight incubation of the first antibody against F4/80 (dilution 1:1000; Abcam) and continued the next day according to the manufacturer’s instructions.

### Lenti-anti and lenti-pre-miR injection

The miR-24-1 pre-miR and miR-ZIP-anti-24 (System Biosciences) were cloned into a human immunodeficiency virus lentiviral vector containing a copGFP reporter with the miRNA precursor under constitutive CMV promoter control. The packaged lentiviral constructs were provided as frozen VSV-G-pseudotyped particles, with their titre (IFU per ml) being defined with the UltraRapid Lentiviral Titer Kit (System Biosciences). The pre-miR-24 (PMIRH24) sequence was: 5′-AATTCGCCCTTGATGGGATTTGCTTCCTGTCACAAATCACATTGCCAGGGATTTCCAACCGACCCTGAGCTCTGCCACCGAGGATGCTGCCCGGGGACGGGGTGGCAGAGAGGCCCCGAAGCCTGTGCCTGGCCTGAGGAGCAGGGCTTAGCTGCTTGTGAGCAGGGTCCACACCAAGTCGTGTTCACAGTGGCTAAGTTCCGCCCCCCAGGCCCTCACCTCCTCTGGCCTTGCCGCCTGTCCCCTGCTGCCGCCTGTCTGCCTGCCATCCTGCTGCCTGGCCTCCCTGGGCTCTGCCTCCCGTGCCTACTGAGCTGAAACACAGTTGGTTTGTGTACACTGGCTCAGTTCAGCAGGAACAGGGGTCAAGCCCCCTTGGAGCCTGCAGCCCCTGCCTTCCCTGGGTGGGCTGATGCTTGGAGCAGAGATGAGGACTCAGAATCAGACCTGTGTCTGGAGGAGGGATGTGGTGGGTGGGGTTGGCTGGGCCCAAATGTGTGCTGCAGGCCCTGATCCCCAACTCTGCAACTGGGGACCCCTGCATGGCCACAGCTCAGGCTGGGCTGTGGTGCCAGCATAGATAGCGGCCGC-3′. The anti-miR-24 (MZIP-24) sequence was: 5′-GATCCGTGGCTCAATTCAGCAGGCACCGCTTCCTGTCAGCTGTTCCTGCTGAACTGAGCCATTTTTGAATT-3′. Anti- and pre-miRs were injected intraperitoneally in 500 ml PBS with 7.6 × 10^7^ IFU per mouse of loaded lentivirus. miR-24-1 modulators were injected one day after AAA induction and compared to a scr-miR control (5′-GTGTAACACGTCTATACGCCCA-3′).

### Double immunofluorescence studies

Primary antibodies for Chi3l1 and CD11b/c or SMA were applied after washing with 10% PBS using conventional IHC dilutions (see above). Secondary antibodies were replaced with antibodies labelled with Alexa Fluor (Invitrogen; dilution 1:250) dye with a maximum excitation at 488 nm (green), and for red with Alexa Fluor 568 (Invitrogen). Images were obtained and analysed by confocal microscopy (Leica SP5, Argon laser).

### *In vitro* studies

Human aortic SMC (hASMCs) and human AoECs were propagated in growth media (SmGM-2 (for hASMC) and EBM-2 (for AoEC); Lonza) with 5% fetal bovine serum (FBS) per standard protocols (Lonza; passage no. 4–5). hASMC were incubated in basal medium (SmBM) for 48 h before treatment/transfection, and other lines were serum-starved overnight before treatment/transfection. RAW 264.7 cells (ATCC) were propagated in DMEM+10% FBS. Cells were treated with human or murine recombinant IL-6 (20 ng ml^−1^; Cell Signaling), IL-4 (5 or 30 ng ml^−1^) and/or CHI3L1/Chi3l1 (30 or 300 ng ml^−1^; R&D Biosystems) for 12 or 24 h. Wells were harvested for RNA analysis at ~90% confluence.

### Luciferase assay

Direct targeting of the CHI3L1–3′UTR by miR-24 was confirmed by transfecting HEK293 cells (ATCC) with Switchgear GoClone luciferase constructs. Cells were transfected in 96-well white-bottomed plates at 40% confluence with plasmids (50 ng per well) containing either empty vector, randomized 3′UTR-positive control or CHI3L1–3′UTR, using FuGene (Switchgear) per manufacturer’s protocol. The cells were then transfected a second time after 24 h with either scr-miR or pre-miR-24 as described above, and incubated for 24 h. Luciferase activity was determined by Lightswitch Assay (Switchgear) as per protocol.

### Isolating peritoneal mouse macrophages

Mice were euthanized by CO_2_, 4 days after injection of Brewer’s thioglycollate (Sigma-Aldrich). A quantity of 5 ml of 4 °C Dulbecco's phosphate-buffered saline (DPBS) were twice injected into the peritoneal cavity of *ApoE*−/− mice with or without ANGII-induced AAA. Macrophages were then withdrawn from the intraperitoneal cavity and put into suspension, spun down (1,500 r.p.m. for 5 min at 4 °C) and 5 ml of RBC lysis buffer was added to the pellet. After incubation and spinning, 1 ml of 37 °C RPMI 1640, 10% FBS and 1% penicillin/streptomycin were added per mouse to the pellet. After counting and dilution (10-fold), cells were plated, analysed and used for further experiments.

### Transfection of cultured cells

Transfection of all different cell types was performed using Lipofectamine RNAiMAX (Invitrogen) reagent, mixed with anti-hsa-miR-24, pre-hsa-miR-24 or scrambled controls (Ambion). For each transfection, 120 pmol of anti- or pre-miR (final concentration at transfection was 50 nM) was diluted in 250 μl Opti-MEM (Gibco). Two hours after transfection, cells were treated with IL-6 as described above and harvested 24 h later. RNA was extracted using TRIzol (Invitrogen). Successful transfection (>80% of all cells) was confirmed by visual fluorescent microscopic analysis and FACS, sorting for Cy3-labelled scr-miR control. Some experiments utilized simultaneous transfection with 150 pmol siRNA (final concentration 50 nM) directed against *CHI3L1/Chi3l1*, with negative siRNA controls (Ambion).

### Western blot analysis

Peritoneal macrophages, RAW 264.7 and hASMCs were homogenized in RIPA lysis buffer. Protein concentrations were determined through the Bicinchoninic Acid assay (Thermo Scientific) according to the manufacturer’s protocol. SDS–polyacrylamide gel electrophoresis (Invitrogen) was performed using 10 μg of protein per well. Western blot was performed with the following primary antibodies: rabbit Chi3l1 (dilution 1:1,000), rabbit Akt (1:500), rabbit p-Akt (1:500), rabbit JNK (1:3,000), rabbit p-JNK (1:3,000) and rabbit IL-10 (1:1,000; all obtained from Abcam), and IL-12 (1:1,000), rabbit ERK (1:3,000) and rabbit p-ERK (1:3,000; all obtained from Cell Signaling), and rabbit β-tubulin (1:1,000; obtained from Sigma-Aldrich; Supplementary Fig. 10).

### Apoptosis assays

Programmed cell death rates were assessed with a commercially available apoptosis assay (Becton Dickinson) in cultured hASMCs. Four different treatment groups were compared with untreated control cells: IL-6 (20 ng ml^−1^), IL-6+anti-miR-24, IL-6+pre-miR-24 and IL-6+scrambled control miR. Cells were treated with 10% H_2_O_2_ in serum-free media for 6 h. Then 1 × 10^5^ cells were harvested and stained with 10 ml fluorescein isothiocyanate Annexin V and 10 ml propidium iodide and FACS sorted within 1 h (BD FACSCaliber, 530 nm (FL1) and >575 nm (FL3)). Assays and FACS analysis were repeated three times.

### Migration assays

VSMC migration in response to CHI3L1 was analysed utilizing a modified Boyden chamber assay^58^. In brief, six-well transwell migration chambers with 8 μm pores (Becton Dickinson) were used. Human aortic SMCs (5 × 10^4^cells per well) were serum-starved in SmBM for 24 h and then plated in the upper chamber in 1 ml of SmBM. SmGM-2 media (2 ml)+recombinant CHI3L1 (0, 3, 30 or 300 ng ml^−1^) were added to the lower chamber, and the cells were allowed to migrate for 24 h at 37 °C. Cells that migrated to the lower chamber were fixed in methanol, stained with 0.1% crystal violet and manually counted in a blinded fashion (eight high-power fields per well).

### Regulation of miR-24 through NF-κB *in vitro*

Transfection of IL-6-treated peritoneal mouse macrophages and RAW 264.7 cells was performed using Lipofectamine RNAiMAX (Invitrogen) reagent, and siRNA targeting either *Rela* (p65) or *Nfkb1* (p50) subunits of the transcription factor NF-κB (Ambion). For each transfection, siRNA was diluted in Opti-MEM for a final transfection concentration of 50 nM. Cells were transfected with Lipofectamine/siRNA 6 h before treatment with IL-6 (20 ng ml^−1^) and incubated for an additional 24 h, followed by harvest and RNA extraction using TRIzol (Invitrogen). Successful knockdown (>75%) was confirmed by qRT–PCR determining expression levels of *Rela* (p65; 5′-TAAGCAGAAGCATTAACTTCTCTGGA-3′; Life Technologies) and *Nfkb1* (p50; 5′-TCAGACGCCATCTATGACAGTAAAG-3′; Life Technologies), as well as decreased nuclear NF-κB activity (ELISA; Active Motif) in control-siRNA- and siRELA-treated cells.

### miRNA cluster regulation with transcriptional arrest

In order to evaluate miR-23b-24-27b cluster processing in response to a pro-aneurysmal inflammatory stimulus, RAW 264.7 cells were treated with actinomycin D (5 μg ml^−1^; Sigma-Aldrich) or DMSO vehicle, with or without recombinant IL-6 (20 ng ml^−1^) over a 24-h time course (samples harvested at 0, 8, 16 and 24 h). Levels of miR-23b, miR-24 and miR-27b throughout the time course were measured utilizing qRT–PCR.

### Human tissue sample acquisition and preparation

Human samples from patients who underwent surgical repair of their AAA, as well as abdominal aortic samples from organ donors, were harvested during surgery (or explantation), snap-frozen and stored at −80 °C.

### Plasma sample acquisition

Blood samples from patients undergoing open repair of their enlarged infrarenal aorta were obtained on the day of surgery after an overnight period of fasting. Specimens were immediately centrifuged, aliquoted and frozen at −80 °C. Samples from patients without visually proven (B-Mode US) AAA disease were part of a screening cohort. Written informed consent was granted from all study participants. For human RNA extraction, 250 μl were used. Murine blood sampling was performed at killing (day 28 in both models) by direct puncture of the left ventricle. RNA was extracted from 50 μl of plasma. CHI3l1/Chi3l1 measurements in human and murine plasma with the appropriate Quantikine ELISA kits were performed according to the manufacturer’s instructions (R&D Biosystems).

### Statistics

If not stated differently, data are presented as mean±s.e.m. Groups were compared using Student’s *t*-test (two-tailed) for parametric data. Normality was tested to ensure that parametric testing was appropriate. When comparing multiple groups, data were analysed by analysis of variance with Bonferroni’s post test. Sequential measurements (AADs at consecutive time points) were analysed by one-way repeated measures analysis of variance. Categorical data were analysed using *χ*^*2*^-test. A value of *P*≤0.05 was considered statistically significant.

### Study approval

Approval for studies on human tissue samples was obtained under informed consent complying with all guidelines and policies of the Stanford University School of Medicine and the Karolinska Institute in accordance with the Declaration of Helsinki. All animal protocols were approved by the Administrative Panel on Laboratory Animal Care at Stanford University ( http://labanimals.stanford.edu/) and followed the National Institutes of Health and United States Department of Agriculture (USDA) Guidelines for Care and Use of Animals in Research. All experiments were performed with 10-week-old male C57BL/6 (PPE model) and 10-week-old male *ApoE*−/− on a C57BL/6 background (ANGII infusion model). Animals were purchased from The Jackson Laboratory.

## Additional information

**Accession codes:** Microarray data were submitted to the National Center for Biotechnology Information's Gene Expression Omnibus under accession code GSE51229.

**How to cite this article:** Maegdefessel, L. *et al.* miR-24 limits aortic vascular inflammation and murine abdominal aneurysm development. *Nat. Commun.* 5:5214 doi: 10.1038/ncomms6214 (2014).

## Supplementary Material

Supplementary Information Supplementary Figures 1-10, Supplementary Tables 1-8, Supplementary Note 1 and Supplementary References
