# Supplementary material for: miR-24 limits aortic vascular inflammation and murine abdominal aneurysm development
Source: Nat Commun. 2014 Oct 31;5:5214. doi: 10.1038/ncomms6214 (PMC4217126; doi:10.1038/ncomms6214)
Supplement: Supplementary Information — Supplementary Figures 1-10, Supplementary Tables 1-8, Supplementary Note 1 and Supplementary References [file ncomms6214-s1.pdf]

Supplementary Figure 1:

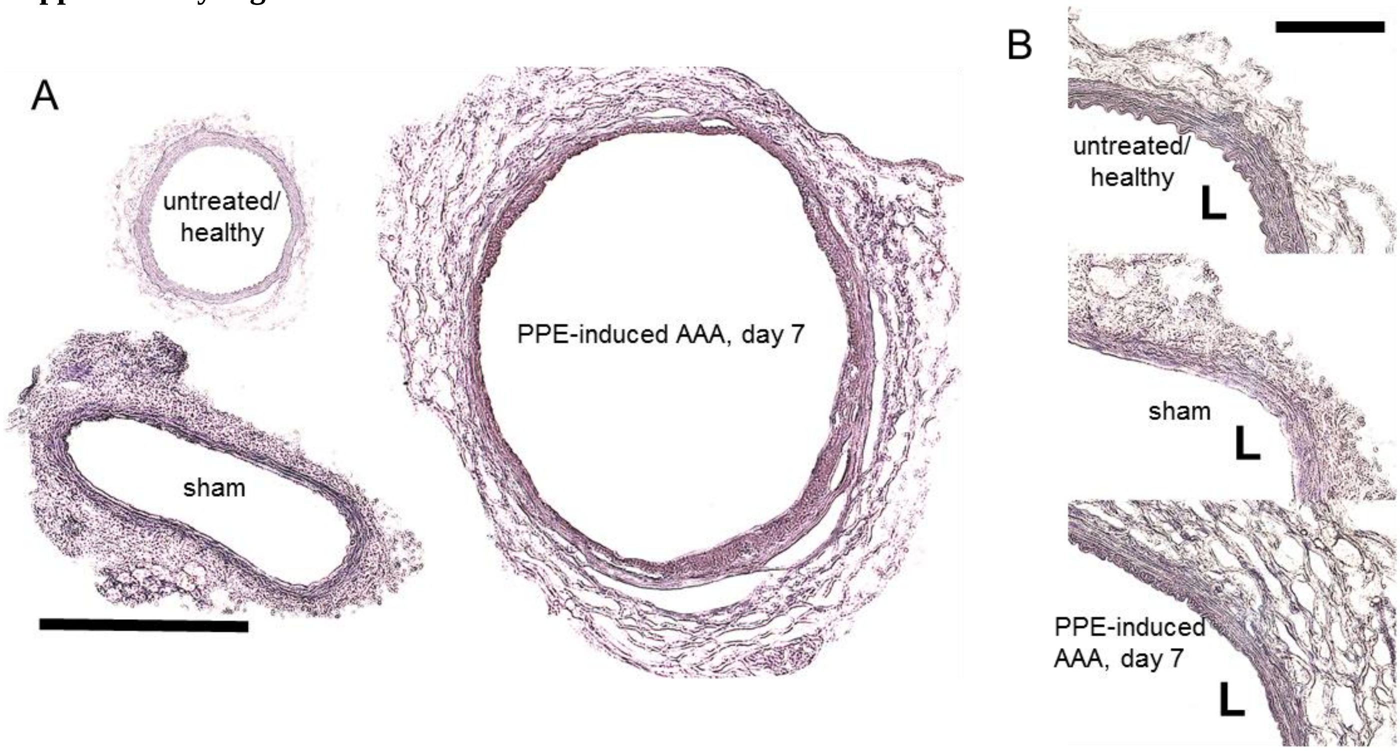

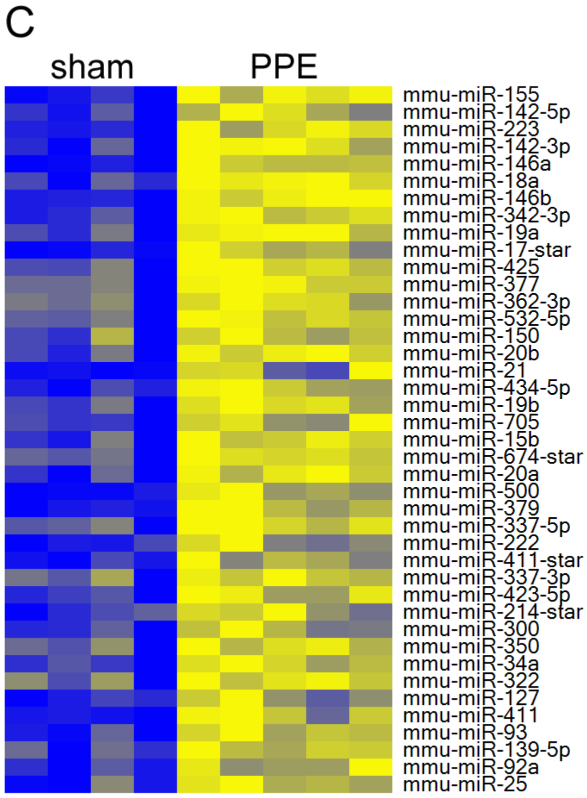

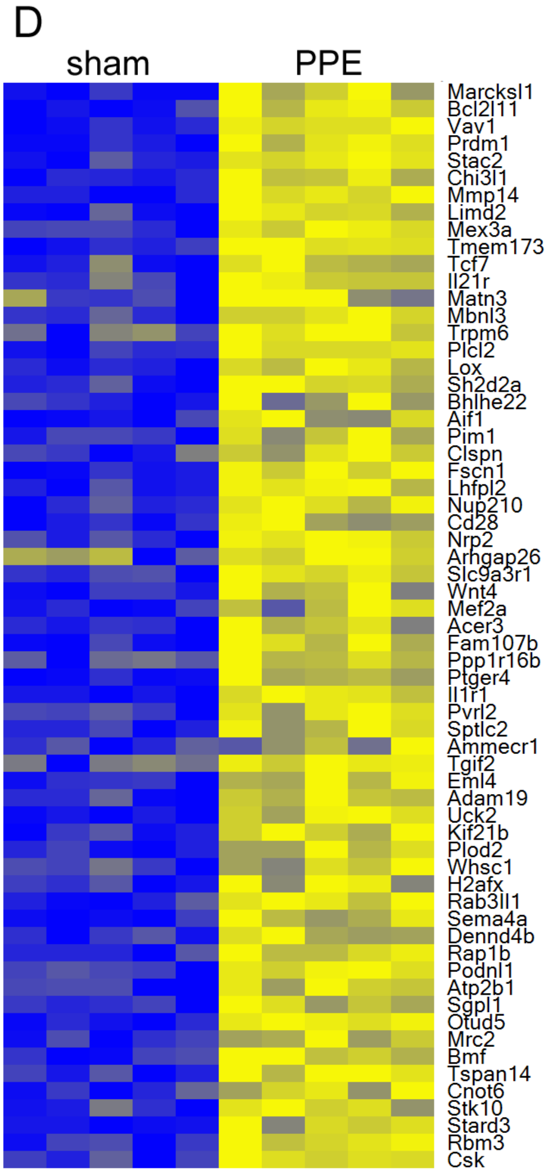

(A) Whole aortic cross-sections stained with Hematoxylin and Eosin (H&E), 7 days after porcine-pancreatic-elastase (PPE)-induced AAA compared to untreated, healthy control aortas and sham (saline-infused; scale bar, 400µm). **(B)** Corresponding images to (A) of cross-sections stained with H&E (scale bar, 50µm, “L” indicates lumen). **(C)** Heat map of significantly up-regulated aortic miRNAs, 7 days after PPE-induction, compared to sham. **(D)** Heat map of significantly up-regulated aortic miR-24 target genes, 7 days after PPE-induction, compared to sham.

Supplementary Figure 2:

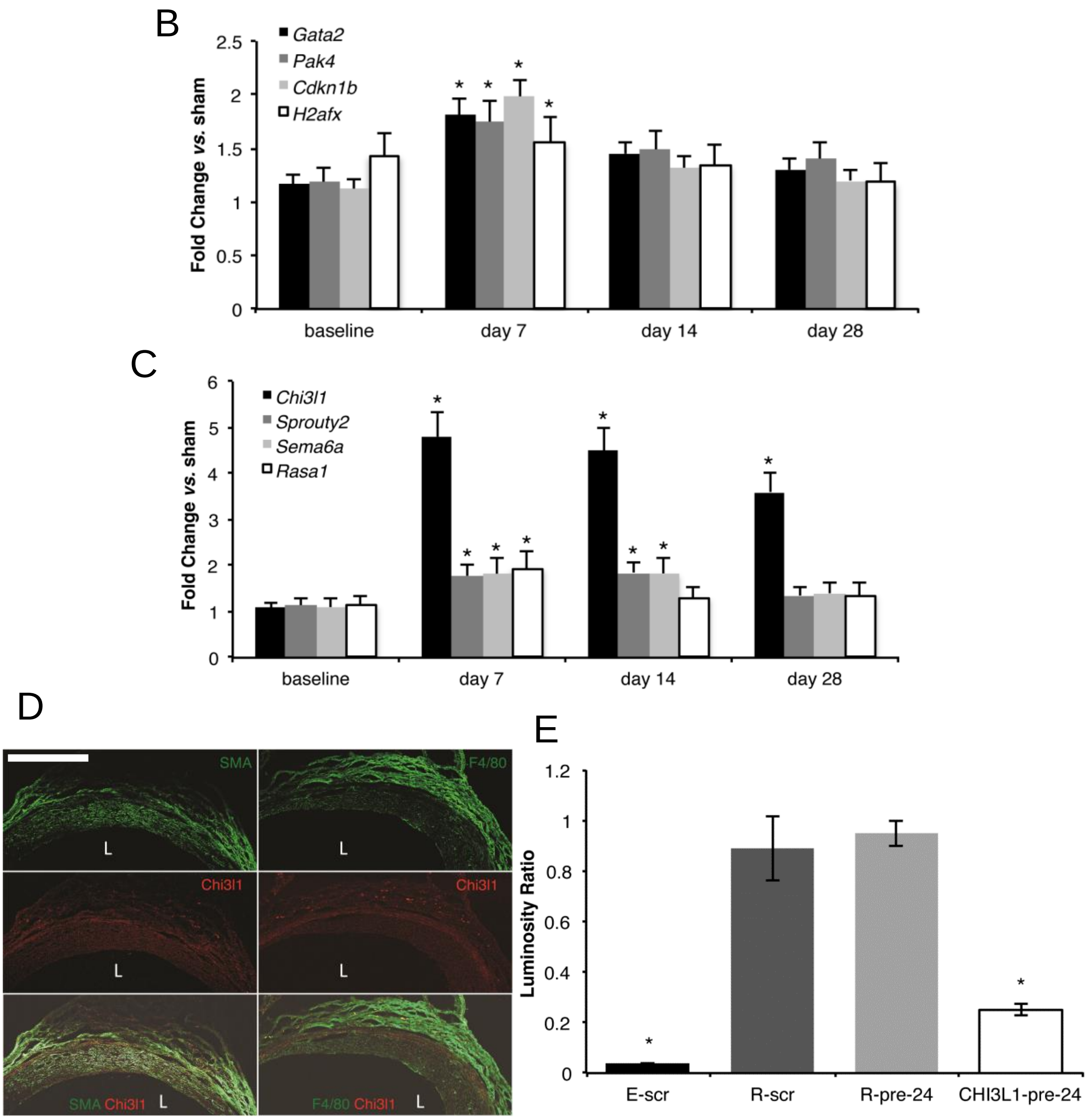

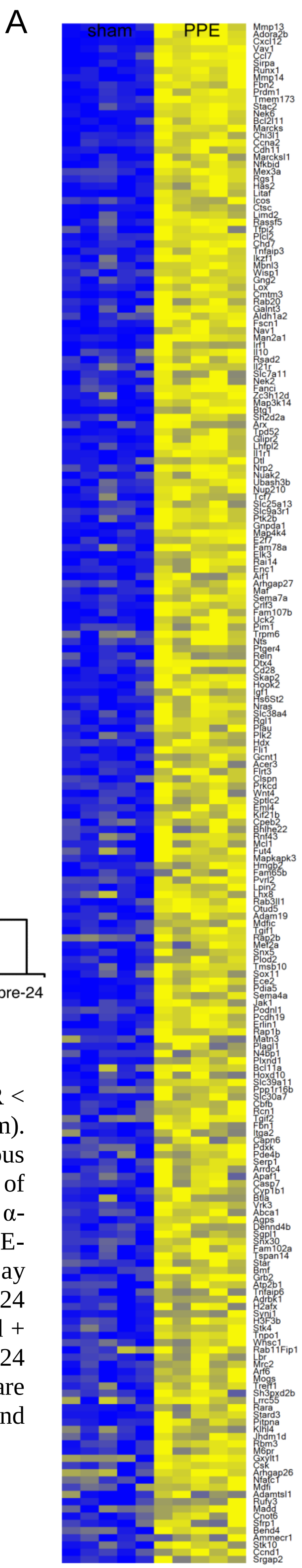

(A) Heatmap showing all significantly up-regulated miR-23b-24-27b target genes (FDR < 1%, fold-change > 1.5) in affected aortic tissue from PPE-induced AAA (Day 7 vs. sham). **(B, C)** Expression of validated miR-24 target genes (from previous studies) at various time-points during PPE-induced AAA expansion (vs. sham), including the expression of *Chi3l1*. **(D)** Double immunofluorescence detection of Chi3l1 (red) and smooth muscle α-actin (SMA) (green, left) or F4-80 (green, right) in aortic sections, 14 days after PPE-induction of AAA ("L" indicates luminal side; scale bar, 50μm). **(E)** Luciferase assay confirming direct suppression of CHI3L1 transcription through 3'UTR binding of miR-24 (E-scr = empty plasmid + scr-miR as negative control, R-scr = random 3'UTR plasmid + scr-miR as positive control, R-pre-24 = random 3'UTR plasmid + pre-24, CHI3L1-pre-24 = CHI3L1 3'UTR plasmid + pre-24). *n* = 4-8 for each group and time point. Data are mean ± SEM. *P<0.05 vs. sham (or R-scr, R-pre-24); analyzed with ANOVA and Bonferroni's post-test.

Supplementary Figure 3:

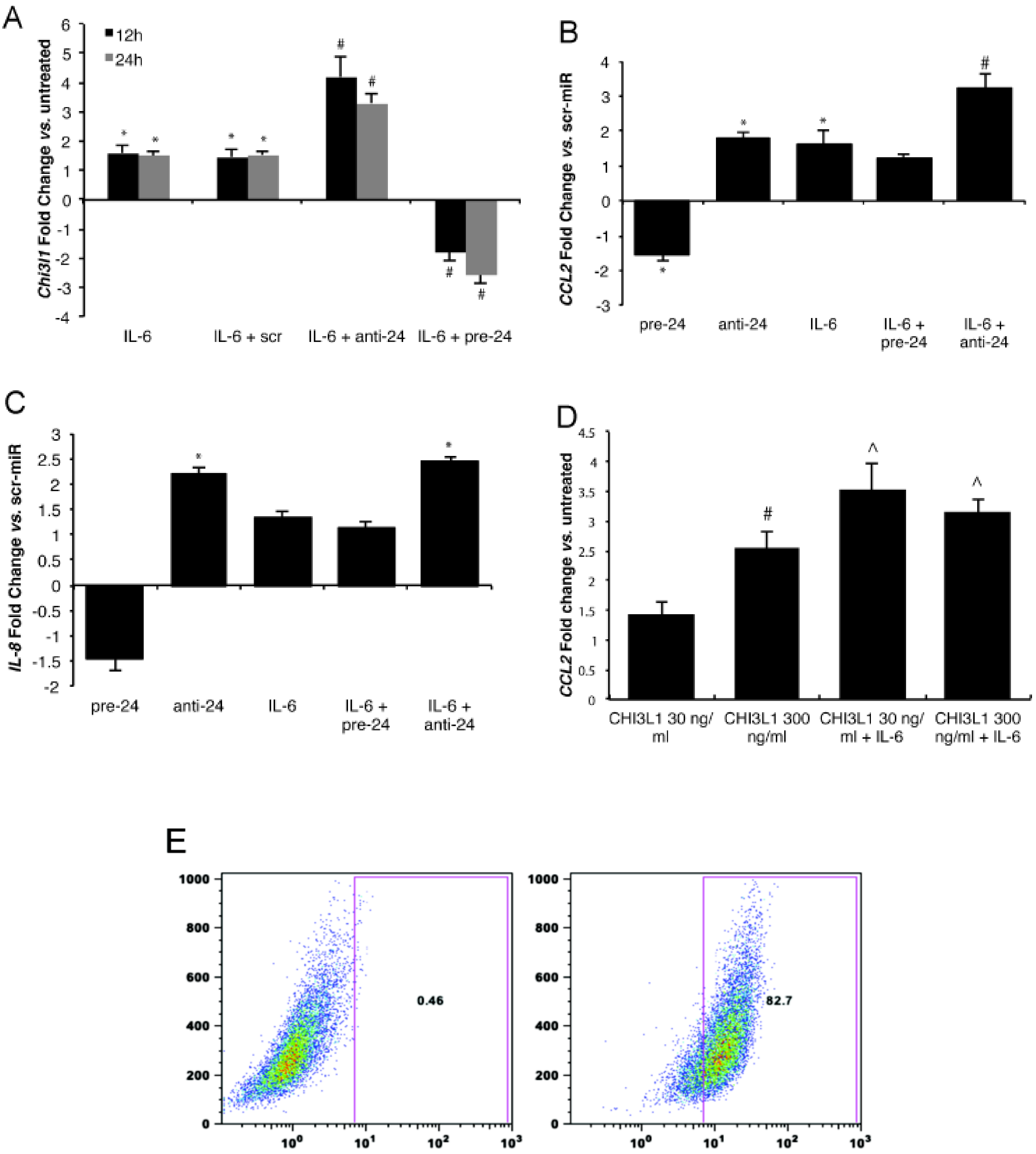

(A) *Chi3l1* expression in interleukin-6 (IL-6)-stimulated (12 or 24 h) and miR-24-modulated peritoneal macrophages. **(B)** *CCL2* expression in IL-6-stimulated and miR-24 modulated hASMC. **(C)** *IL8* expression in IL-6 stimulated and miR-24 modulated hASMC. **(D)** *CCL2* expression in CHI3L1-stimulated hASMC ± pre-stimulation with IL-6 (16 h). **(E)** FACS analysis of C3-labeled, scr-miR-transfected, peritoneal macrophages. First image displays un-transfected control cells, and second image indicates 82.7% transfection efficiency using scr-miR. All experiments were run in triplicate. Data are mean ± SEM. **P*<0.05 *vs.* untreated or scr-miR, $^{\#}$*P*<0.05 *vs.* untreated + scr-miR or *vs.* anti-/pre-24 (in Suppl. Fig. 2C and 2D) or *vs.* untreated + CHI3L1 30ng/ml (Suppl. Fig. 3E), $^{\wedge}$*P*<0.05 *vs.* untreated + CHI3L1 treatment alone, analyzed with ANOVA and Bonferroni's post-test.

Supplementary Figure 4:

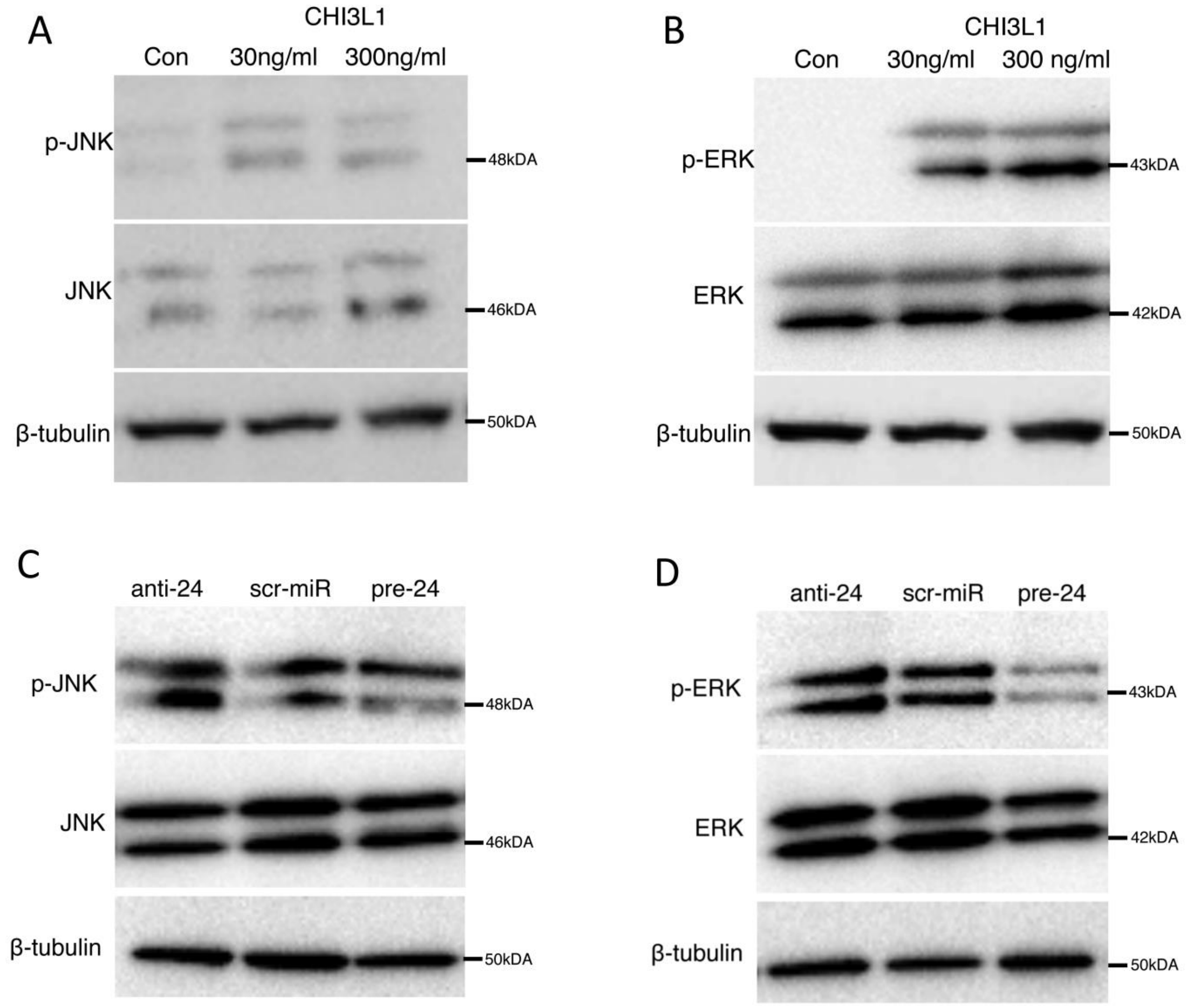

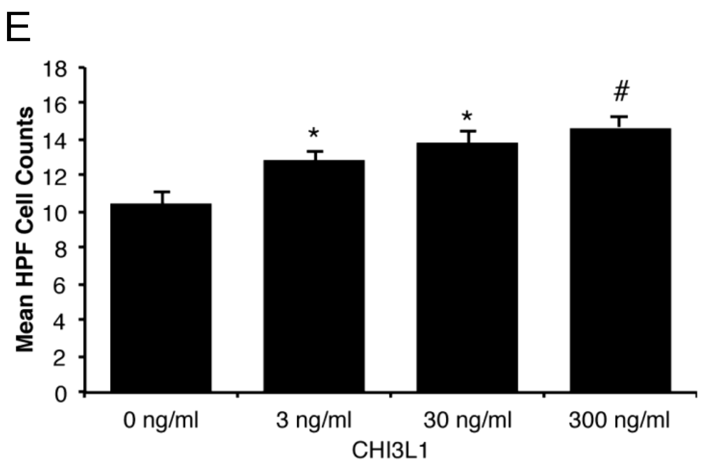

(A) Protein expression of total JNK (JNK) and phospho-JNK (p-JNK) in low-dose (30ng/ml) and high dose (300ng/ml) CHI3L1 stimulated human aortic smooth muscle cells (hASMC) with β-tubulin loading control. **(B)** Protein expression of total ERK (ERK) and phospho-ERK (p-ERK) in low-dose (30ng/ml) and high dose (300ng/ml) CHI3L1 stimulated hASMC (β-tubulin loading control). **(C)** Protein expression of total JNK (JNK) and phospho-JNK (p-JNK) in miR-24 modulated hASMC (β-tubulin loading control).
(D) Protein expression of total ERK (ERK) and phospho-ERK (p-ERK) in miR-24 modulated hASMC (β-tubulin loading control). **(E)** Modified Boyden chamber migration assay in CHI3L1-stimulated hASMC at various doses. High power field = HPF. Data are mean ± SEM. $^{*}P<0.05$ *vs.* control/scr-miR, $^{\#}P<0.05$ *vs.* all other treatments in the same experiment, analyzed with ANOVA and Bonferroni's post-test.

Supplementary Figure 5:

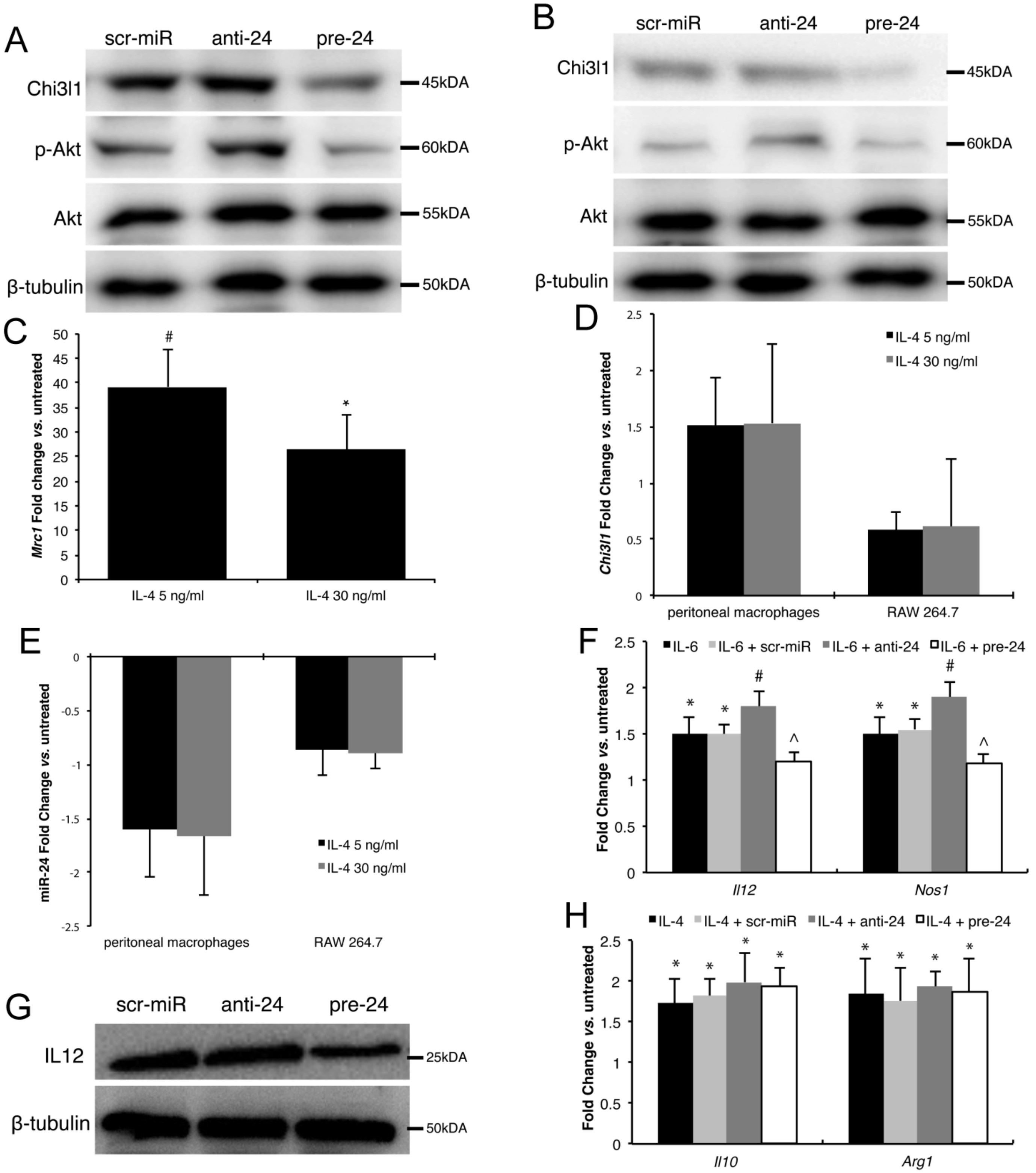

(A) Protein expression of Chi3l1 and its down-stream effector p-Akt in IL-6-stimulated and miR-24-modulated peritoneal macrophages (vs. scr-miR). **(B)** Protein expression of Chi3l1 and its down-stream effector p-Akt in IL-6-stimulated and miR-24 modulated RAW 264.7 cells. **(C)** *Mrc1* expression in RAW 264.7 cells stimulated with interleukin-4 (IL-4). **(D)** *Chi3l1* expression in RAW 264.7 and peritoneal macrophages, stimulated with IL-4. **(E)** miR-24 expression in RAW 264.7 and peritoneal macrophages, stimulated with IL-4. **(F)** *Il12* and *Nos1* expression in IL-6 stimulated and miR-24 modulated peritoneal macrophages. **(G)** Protein expression of IL12 in miR-24 modulated hASMC with β-tubulin loading control. **(H)** *Il10* and *Arg1* expression in IL-4 stimulated and miR-24 modulated peritoneal macrophages. All experiments were run in triplicate. Data are mean ± SEM. $^{*}P<0.05$ *vs.* control/scr-miR, $^{\#}P<0.05$ *vs.* all other treatments in the same experiment, analyzed with ANOVA and Bonferroni's post-test.

Supplementary Figure 6:

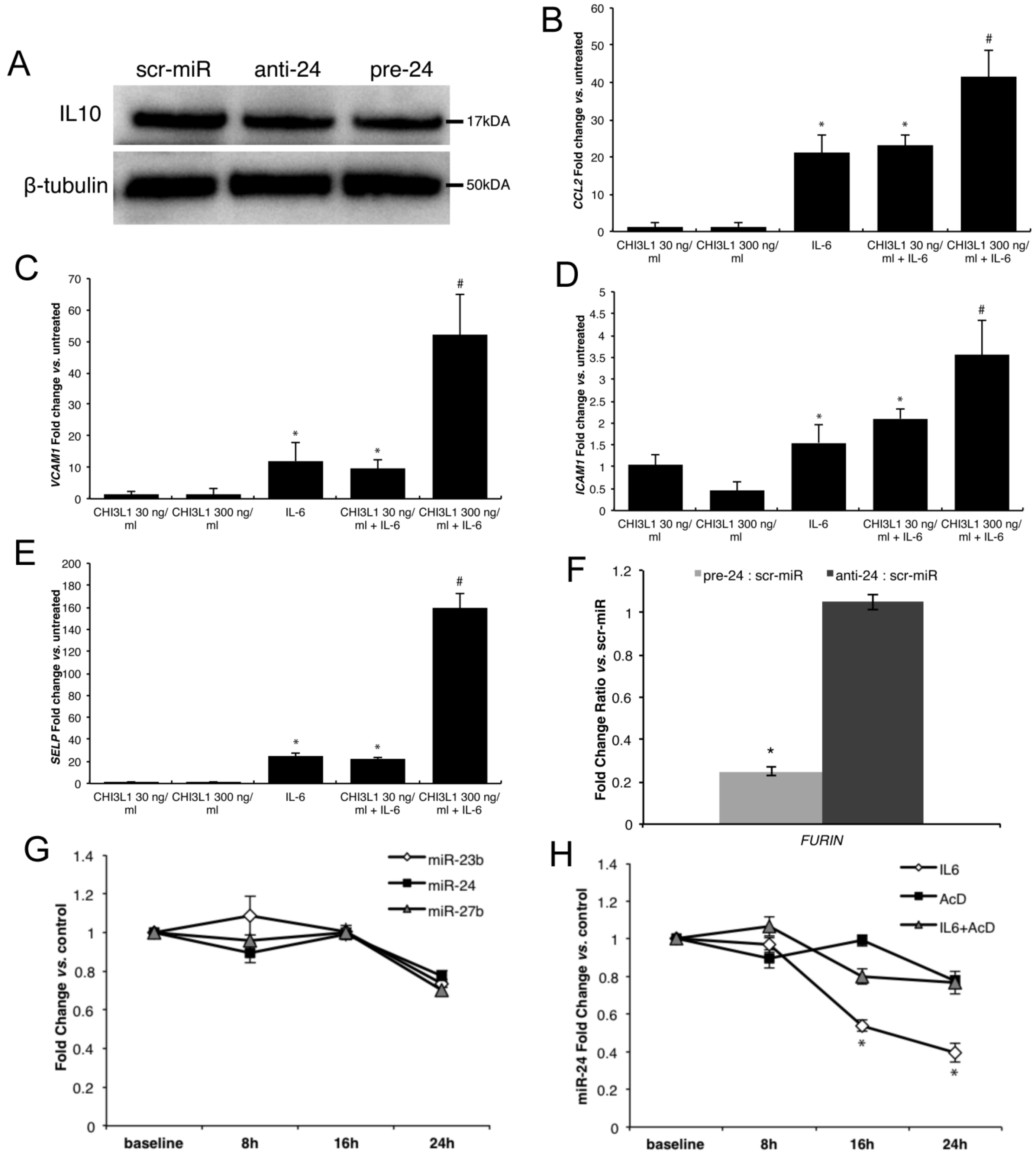

(A) Protein expression of IL10 in miR-24 modulated hASMC with β-tubulin loading control. **(B)** *CCL2* expression in CHI3L1-stimulated human aortic endothelial cells (hAEC) ± IL-6. **(C)** *VCAM1* expression in CHI3L1-stimulated hAEC ± IL-6. **(D)** *ICAM1* expression in CHI3L1-stimulated hAEC ± IL-6. **(E)** *SELP* expression in CHI3L1-stimulated hAEC ± IL-6. miR-24 expression in miR-24-modulated, porcine-pancreatic-elastase(PPE)-induced AAA. **(F)** *FURIN* expression in miR-24 modulated hASMCs. **(G)** miR-23b-24-27b fold change vs. vehicle- treated control in IL-6 stimulated RAW 264.7 cells at baseline, and after 8h, 16h and 24h. **(H)** miR-24 degradation/processing (displayed as fold change vs. vehicle-treated control) in IL-6 stimulated and actinomycin D (AcD) treated RAW 264.7 cells over time. All experiments were run in triplicate. Data are mean ± SEM. $^*P<0.05$ *vs.* control or untreated, $^{\#}P<0.05$ *vs.* all other treatments in the same experiment, analyzed with ANOVA and Bonferroni's post-test.

Supplementary Figure 7:

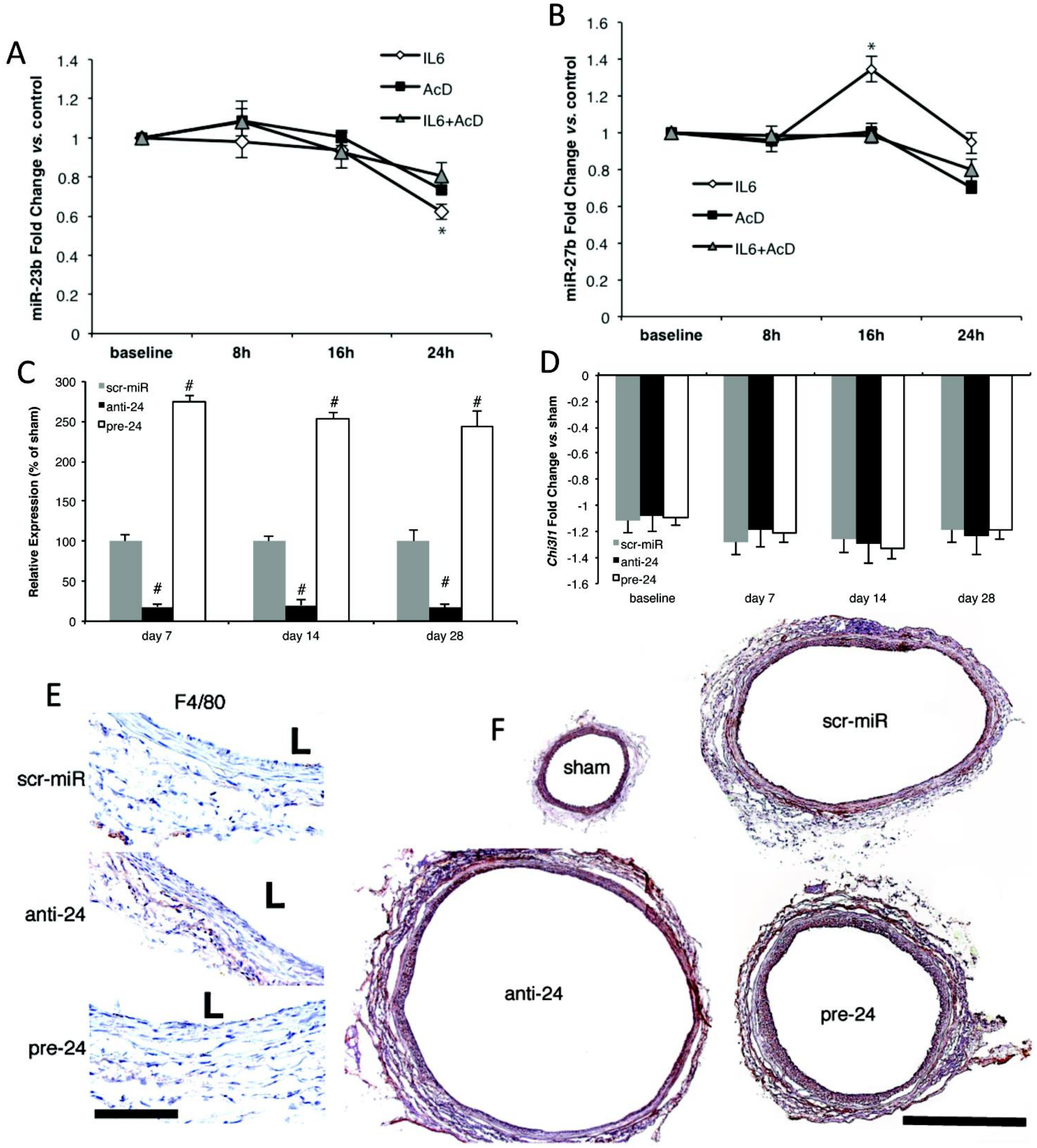

(A) miR-23b degradation/processing (displayed as fold change vs. vehicle-treated control) in IL-6 stimulated and actinomycin D (AcD) treated RAW 264.7 cells over time. **(B)** miR-27b degradation/processing (displayed as fold change vs. vehicle-treated control) in IL-6 stimulated and actinomycin D (AcD) treated RAW 264.7 cells over time. **(C)** Relative miR-24 expression (% of sham) in miR-24-modulated, PPE-induced AAA. **(D)** *Chi3l1* expression in suprarenal/non-aneurysmal PPE-induced and miR-24 modulated AAA over time. **(E)** F4/80 (macrophage) staining in miR-24 modulated mice with PPE-induced AAA (day 14; "L" indicates luminal side; scale bar, 50μm). **(F)** Whole murine aortic cross-sections (scale bar, 400μm) stained for SMA in miR-24 modulated with PPE-induced AAA (day 14; corresponding detailed images are presented in Figure 5A). *n* = 4-6 mice for each group. Data are mean ± SEM. #P<0.05 vs. scr-miR and sham, analyzed with ANOVA and Bonferroni's post-test.

Supplementary Figure 8:

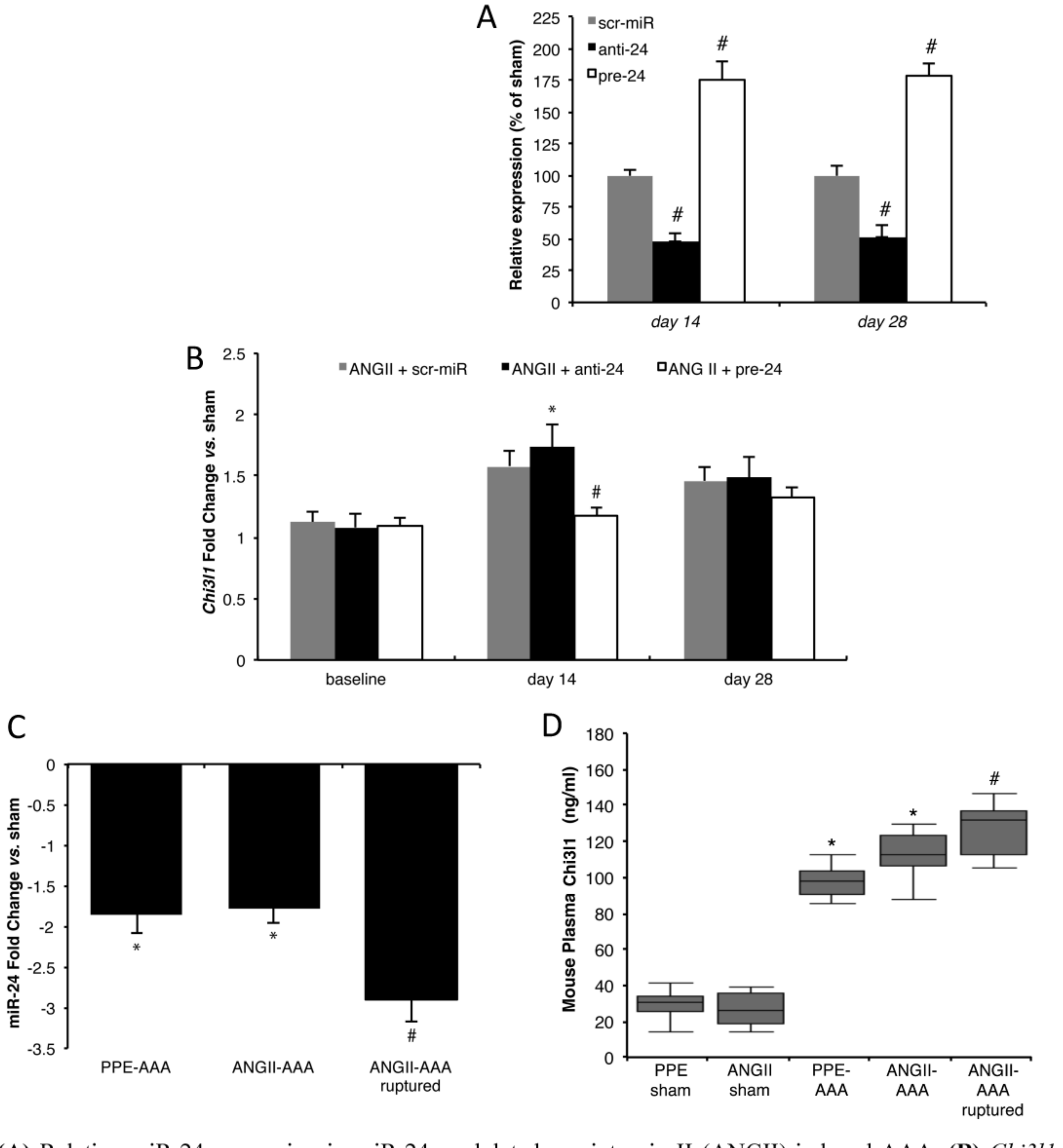

(A) Relative miR-24 expression in miR-24-modulated, angiotensin II (ANGII)-induced AAA. **(B)** *Chi3l1* expression in infrarenal ANGII-induced and miR-24 modulated AAA over time. **(C)** miR-24 expression (displayed as fold change vs. sham) in plasma from mice with either PPE-induced AAA (n = 11), ANGII-induced AAA (n = 9) or ANGII-induced AAA with covered rupture or dissection (n = 7) detected with B-mode ultrasound). **(D)** Chi3l1 levels (measured by ELISA) in serum from mice with either PPE-induced AAA (n = 8), ANGII-induced AAA (n = 8) or ANGII-induced AAA with covered rupture or dissection (n = 5) detected with B-mode ultrasound) compared to PPE and ANGII sham. For experiments in Supplemental Figure 8A and 8B: n = 4-6 mice for each group. Data are mean ± SEM. *P<0.05 *vs.* sham, $^{\#}P$<0.05 vs. all other treatment groups (or vs. PPE-induced AAA in Figure D), analyzed with ANOVA and Bonferroni's post-test.

Supplementary Figure 9: Proposed mechanism

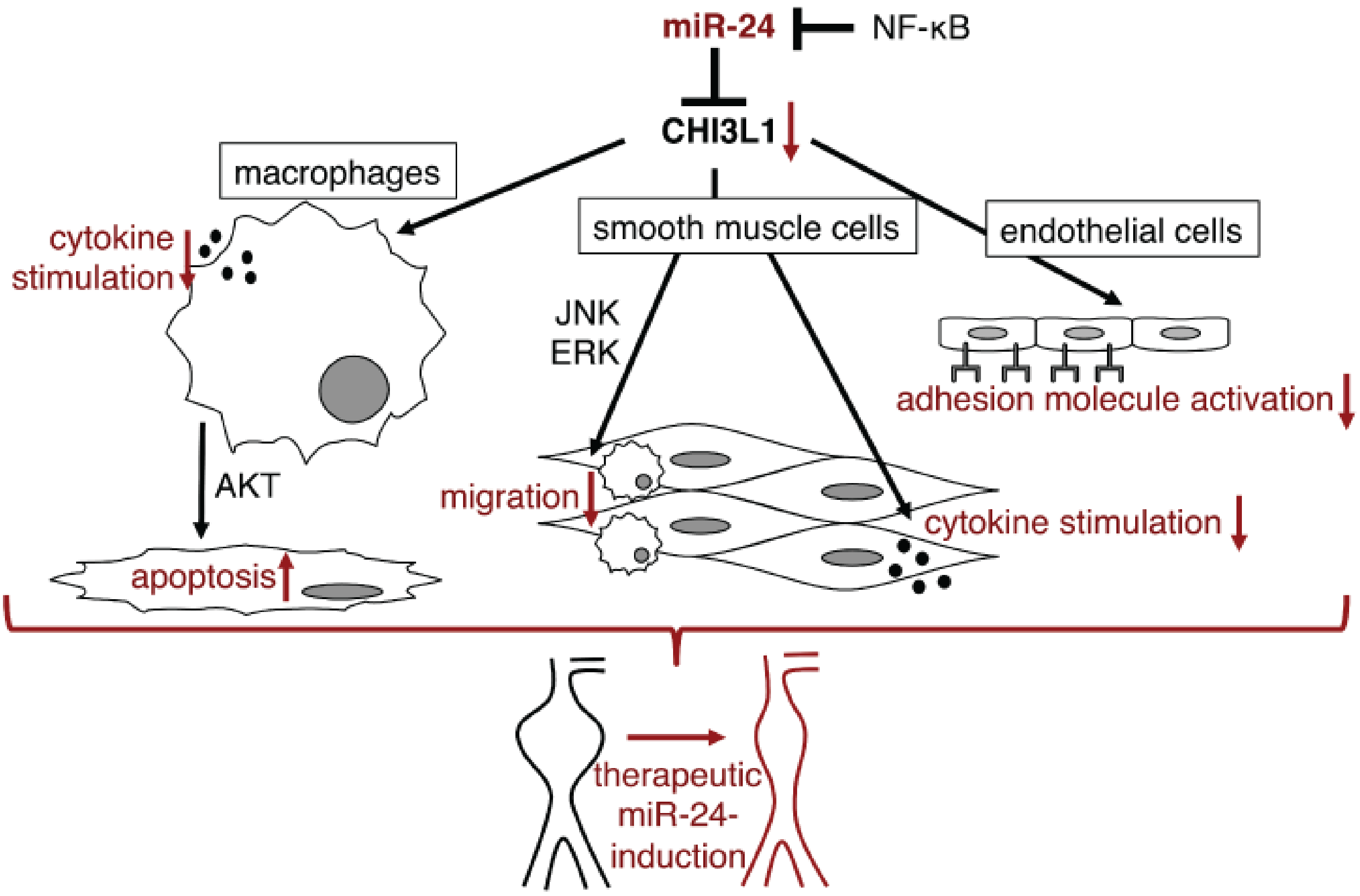

Supplementary Figure 10:

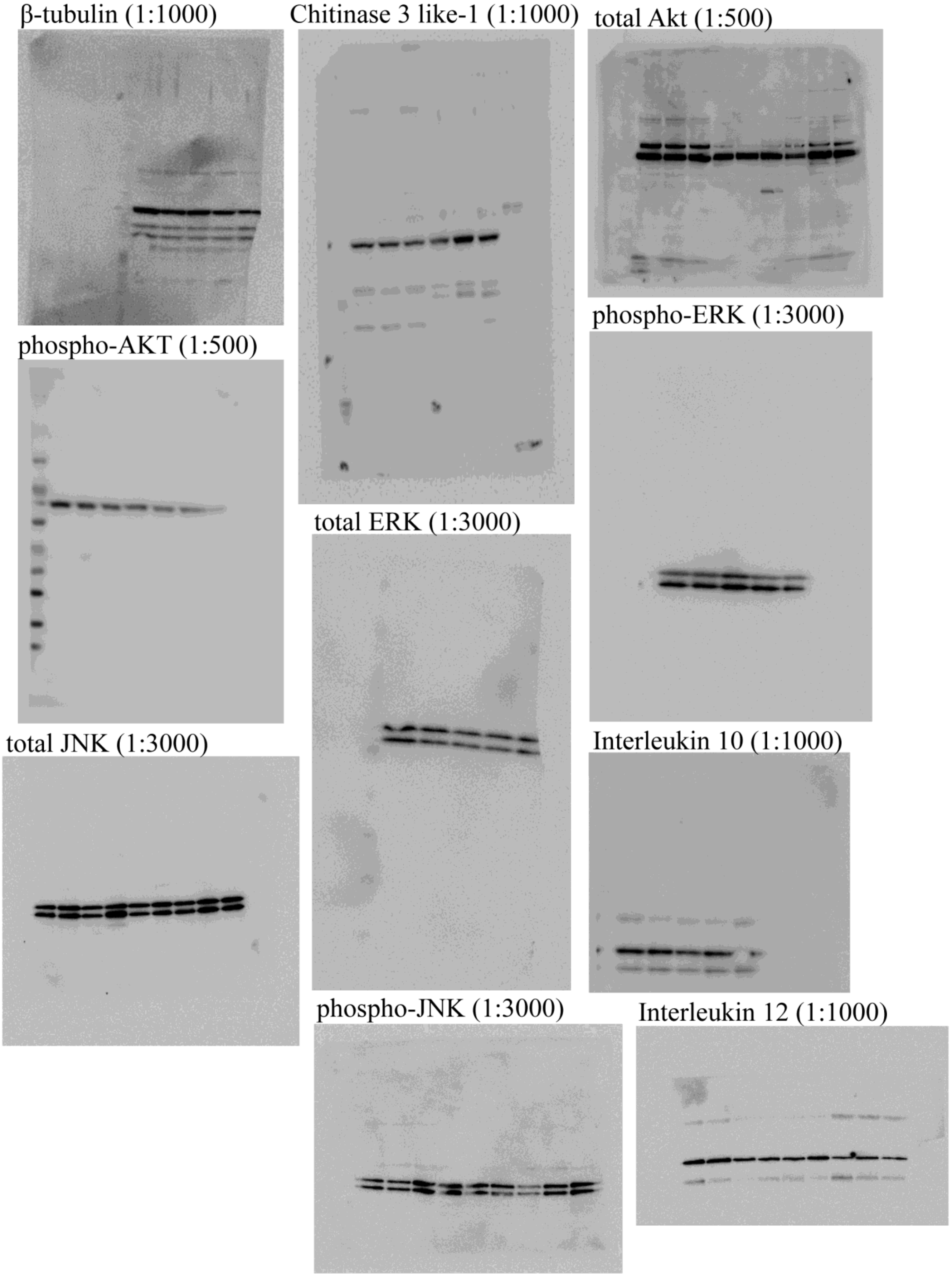

Un-cropped Western blots for all antibodies (with dilutions) utilized in our manuscript. Technical details are described in the Methods.

Supplementary Tables

Table 1: Absolute ultrasound diameter measurements in the PPE-model

	**baseline**	**day 3**	**day 7**	**day 14**	**day 21**	**day 28**
**sham**	0.63 ± 0.003	0.71 ± 0.02	0.75 ± 0.02	0.78 ± 0.007	0.77 ± 0.07	0.77 ± 0.09
**PPE**	0.62 ± 0.004	0.75 ± 0.03	*0.86 ± 0.04**	*1.05 ± 0.03**	*1.12 ± 0.08**	*1.13 ± 0.06**

Absolute measurements (mean ± SEM; in mm) of the abdominal aortic diameter using B-mode ultrasound from the porcine-pancreatic-elastase (PPE) induction model from baseline until day 28. Absolute measurements correspond to Figure 1A (presented as AAD *vs.* baseline in %) in the manuscript. $P < 0.05$ is considered significant vs. same- day sham, using *Student's t-test* for parametric measures *(*)*.

Table 2: Absolute ultrasound diameter measurements in the ANGII-model

	**baseline**	**day 7**	**day 14**	**day 28**
**sham**	1.10 ± 0.12	1.14 ± 0.13	1.18 ± 0.15	1.13 ± 0.12
**ANGII**	1.11 ± 0.1	1.28 ± 0.12	*1.76 ± 0.17**	*2.01 ± 0.21**

Absolute measurements (mean ± SEM; in mm) of the abdominal aortic diameter using B-mode ultrasound in the angiotensinII-AAA induction model from baseline until day 28. Absolute measurements correspond to Figure 2A (presented as AAD *vs.* baseline in %). $P < 0.05$ is considered significant vs. same-day sham, using *Student's t-test* for parametric measures *(*)*.

Table 3: Blood pressure measurements in mice with AAA

	**systolic (mmHg)**	**diastolic (mmHg)**	**p-value**
**PPE (*n*=6)**	112 ± 11	81 ± 8	
**sham (for PPE; *n*=6)**	109 ± 8	83 ± 7	0.248
**ANGII (*n*=6)**	*145 ± 9**	92 ± 8	
**sham (for ANGII; *n*=6)**	116 ± 8	90 ± 9	*0.033**

Blood pressure measurements (systolic/diastolic) in the porcine-pancreatic-elastase (PPE) and angiotensin II (ANGII)-AAA induction models (day 14). Data are mean ± SEM. $P < 0.05$ is considered significant, using *Student's t-test* for parametric measures (to compare PPE vs. sham and ANGII *vs.* sham*; *)*.

Table 4: Absolute ultrasound diameter measurements in the PPE-model after miR-24 modulation

	baseline	day 3	day 7	day 14	day 21	day 28
**PPE anti-24**	0.64 ± 0.005	0.84 ± 0.015	0.95 ± 0.024	*1.19 ± 0.033**	*1.31 ± 0.028**	*1.32 ± 0.028**
**PPE scr-miR**	0.64 ± 0.011	0.83 ± 0.012	0.90 ± 0.012	1.03 ± 0.022	1.12 ± 0.02	1.11 ± 0.019
**PPE pre-24**	0.63 ± 0.01	0.82 ± 0.013	0.87 ± 0.016	*0.95 ± 0.017**	*1.01 ± 0.015**	*1.01 ± 0.015**

Absolute measurements (mean ± SEM; in mm) of the abdominal aortic diameter using B-mode ultrasound from the porcine-pancreatic-elastase (PPE) induction model from baseline until day 28 in anti-/pre-24 and scr-miR mice with AAA. Absolute measurements correspond to Figure 4B (presented as AAD *vs.* baseline in %) in the manuscript. *P < 0.05* is considered significant, using one-way ANOVA with Bonferroni's post-test *(*)*.

Table 5: Absolute ultrasound diameter measurements in the ANGII-model after miR-24 modulation

	baseline	day 7	day 14	day 28
**ANGII anti-24**	1.11 ± 0.03	1.36 ± 0.04	*1.94 ± 0.05**	*2.25 ± 0.04**
**ANGII scr-miR**	1.09 ± 0.04	1.35 ± 0.06	1.73 ± 0.05	1.94 ± 0.05
**ANGII pre-24**	1.13 ± 0.03	1.34 ± 0.05	*1.60 ± 0.04**	*1.79 ± 0.05**

Absolute measurements (mean ± SEM; in mm) of the abdominal aortic diameter using B-mode ultrasound in the angiotensin II (ANGII)-AAA induction model from baseline until day 28 in anti-/pre-24 and scr-miR mice with AAA. Absolute measurements correspond to Figure 6A (presented as AAD *vs.* baseline in %) in the manuscript. *P < 0.05* is considered significant, using one-way ANOVA with Bonferroni's post test *(* at day 14 only for anti-21 vs. pre-24).*

Table 6: Blood pressure measurements in miR-24 modulated mice

	systolic (mmHg)	diastolic (mmHg)	p-value
**scr-miR (*n*=4)**	106 ± 7	81 ± 1.5	
**anti-24 (*n*=4)**	105 ± 7	80 ± 2	0.974
**pre-24 (*n*=4)**	106 ± 9	79 ± 2	0.275

Blood pressure measurements (systolic/diastolic) in otherwise-untreated miR-24-modulated mice (anti- and pre-24 compared to scr-miR). Data are mean ± SEM. *P < 0.05* is considered significant, using one-way ANOVA with Bonferroni's post-test.

Table 7: Pathway analysis of transcriptional profiling of in porcine-pancreatic-elastase (PPE)-induced abdominal aortic aneurysm (AAA) post-miR-24 modulation

	sham>scr-miR/PPE	sham>pre-24/PPE	overlap
**GO Pathways**			
Oxidation reduction	135	28	15
Cell adhesion	120	32	26
Steroid metabolism	37	8	4
**KEGG pathways**			
Focal adhesion	58	11	10
VSMC contraction	40	8	6
ECM-receptor interaction	26	9	9

	scr-miR/PPE>sham	pre-24/PPE>sham	overlap
**GO Pathways**			
Positive regulation of immune system	104	14	14
Induction of apoptosis	60	8	6
**KEGG pathways**			
Cytokine-cytokine receptor interaction	104	12	12
Leukocyte transendothelial migration	42	6	6
Toll-like receptor signaling	51	7	7

Scr-miR (scrambled-miR-injected mouse with PPE-induced AAA); pre-24 (pre-miR-24 transduced mice with PPE-induced AAA); VSMC (vascular smooth muscle cells); ECM (extracellular matrix). Numbers of differentially regulated genes in each pathway at day 7 post-AAA induction shown. See Supplementary Note 1 for details.

Table 8: Baseline patient characteristics for plasma measurement of the expression of miR-24 and YKL-40 levels

	Controls	Small AAA	Large AAA	PVOD	p-value
***n* =**	52	54	51	22	-
**AAD (in mm)**	≤ 35	*57 ± 7**	*85 ± 14**	≤ 35	*≤0.001**
**Age (in years)**	65 ± 3	65 ± 2	65 ± 3	66 ± 4	0.149
**Male Sex (in %)**	100	100	100	100	-
**Smokers (in %)**	93	91	90	91	1.000
**eGFR**	84.3 ± 11.2	79.5 ± 12.5	79.3 ± 10.6	81.9 ± 9.1	0.086
**Triglycerides (mmol/l)**	2.3 ± 0.4	2.5 ± 0.5	2.2 ± 0.4	2.1 ± 0.7	0.777
**Bilirubin (µmol/L)**	11.9 ± 2.3	10.9 ± 2.4	11.3 ± 2.2	11.4 ± 2.2	0.164
**ASAT (U/L)**	26.9 ± 7.6	28.3 ± 9.3	29.6 ± 10.5	30.2 ± 7.8	0.367
**HbA1c (rel. %)**	5.8 ± 0.4	5.7 ± 0.5	5.9 ± 0.4	6.0 ± 0.2	0.097
**NSAID (in %)**	19	20	22	*90**	*≤0.001**
**ACEI/ARB (in %)**	79	78	81	82	0.934
**Beta blocker (in %)**	65	72	74	*27**	*0.001**
**Statins (in %)**	85	89	86	86	0.809

Data are mean ± standard deviation (SD), number (*n*) of individuals and percentages (%). $P < 0.05$ is considered significant (*), using ANOVA with Bonferroni's post test for parametric data with normal distribution. For categorical, non-parametric data (smoking status and medication), Chi-squared test (χ^2) was utilized. AAA (abdominal aortic aneurysm); AAD (abdominal aortic diameter measured by ultrasound); ACEI (angiotensin converting enzyme inhibitor); ARB (angiotensin II receptor blocker); ASAT (aspartate aminotransferase); eGFR (estimated glomerular filtration rate), HbA1c (glycosylated hemoglobin); PVOD (peripheral vascular occlusive disease); NSAID (non-steroidal anti-inflammatory drug).

Supplementary Note 1

miR-24 modulation effects on gene expression in murine AAA (expanded)

We collected infra-renal aortic tissue from miRNA-modulated and saline-infused control mice 7 days after infusion, and hybridized isolated RNA to Agilent mouse whole genome microarrays. Expression analysis identified numerous up-regulated and down-regulated genes (1,996 and 1,200 respectively, at FDR<1%) in scr-miR-treated PPE-induced AAA vs. scr-miR-treated saline-infused control mice.

Ontologic pathway enrichment analysis of significant gene lists at 7-days of treatment focusing on KEGG pathways and GO Biological Processes was performed as previously described[1]. Differentially regulated PPE-scr-miR genes described above yielded similar enriched pathways to those seen previously in ANGII-induced aneurysms, featuring pathways related to immune responses, matrix remodeling, and cell signaling. Up-regulated (with PPE-induced aneurysm) pathways included cell cycling, apoptosis, toll-like receptor signaling, and regulation of the NF-κB cascade, as well as MAPK and p53 signaling (Supplemental Table 7). As in our previous work, we identified broad increases in genes associated with immune system activation (e.g. macrophage, T-cell, B-cell and natural killer responses), lymphocyte proliferation and differentiation, and numerous cytokines and cytokine receptors. For example, 104 genes in the GO category “positive regulation of immune system process” were up-regulated in scr-miR aorta when compared with saline control. As expected, *Chi3l1* was up-regulated (4.5-fold) with aneurysm formation.

Down-regulated gene responses to treatment also mimicked to some extent those seen previously with ANGII-aneurysm formation, with enrichment of SMC contractile genes, focal adhesion, insulin signaling, and numerous “metabolism” pathways such as oxidative phosphorylation and amino acid/lipid metabolism[1].

Notably, ontology of anti-24-modulated PPE-infused gene expression was essentially indistinguishable from that seen in scr-miR-treated PPE-infused mice. Further, when anti-24 PPE-infused expression was compared with saline-infused sham-operated controls, the gene numbers and differentially regulated pathways were very similar to those obtained by comparing scr-miR PPE-mice with saline-infused sham controls. *Chi3l1* was further up-regulated with anti-24 treatment.

In contrast, gene expression in pre-24-modulated aortic tissue was much harder to distinguish from sham controls, with only 208 unique genes higher in pre-24 than sham, and 630 genes lower at FDR < 1%. While some of the enriched pathways

were similar overall to those found in scr-miR-PPE *vs.* sham, the numbers of genes within each categories were much smaller, including for example only 14 “positive regulation of immune system process” genes up-regulated with pre-24. Down-regulated metabolism pathways were also less well represented in pre-24 modulated aortas. *Chi3l1* gene expression was undetectable by microarray in pre-24-treated aortas, confirming our qRT-PCR results. Partial lists of enriched KEGG and GO categories with a direct comparison between significant scr-miR and pre-miR pathways are in Supplemental Table 7.

Reference

1. Spin, J.M., *et al.* Transcriptional profiling and network analysis of the murine angiotensin II-induced abdominal aortic aneurysm. *Physiological genomics* **43**, 993-1003 (2011).
